# *NTRK1* fusions for the therapeutic intervention of Korean patients with colon cancer

**DOI:** 10.18632/oncotarget.6724

**Published:** 2015-12-22

**Authors:** Do Youn Park, Chan Choi, Eunji Shin, Jae Hyuk Lee, Chae Hwa Kwon, Hong-Jae Jo, Hyeong-Rok Kim, Hyun Sung Kim, Nahmgun Oh, Ji Shin Lee, Ok Ku Park, Eok Park, Jonghoon Park, Jong-Yeon Shin, Jong-Il Kim, Jeong-Sun Seo, Hee Dong Park, Joonghoon Park

**Affiliations:** ^1^ Department of Pathology, Pusan National University Hospital and Pusan National University School of Medicine, and BioMedical Research Institute, Pusan National University Hospital, Busan, Republic of Korea; ^2^ Department of Surgery, Pusan National University Hospital and Pusan National University School of Medicine, and BioMedical Research Institute, Pusan National University Hospital, Busan, Republic of Korea; ^3^ Department of Pathology, Chonnam National University Hwasun Hospital, Hwasun-gun, Jeollanam-do, Republic of Korea; ^4^ Department of Surgery, Chonnam National University Hwasun Hospital, Hwasun-gun, Jeollanam-do, Republic of Korea; ^5^ Genomic Medicine Institute (GMI), Medical Research Center, Seoul National University, Seoul, Republic of Korea; ^6^ Department of Biochemical and Molecular Biology, Seoul National University College of Medicine, Seoul, Republic of Korea; ^7^ Macrogen Inc., Seoul, Republic of Korea; ^8^ LG Life Sciences Ltd., R&D Park, Daejeon, Republic of Korea

**Keywords:** NTRK1 fusion, Korean colon cancer, RNA-seq, predictive biomarker, targeted therapy

## Abstract

The identification and clinical validation of cancer driver genes are essential to accelerate the translational transition of cancer genomics, as well as to find clinically confident targets for the therapeutic intervention of cancers. Here we identified recurrent *LMNA-NTRK1* and *TPM3-NTRK1* fusions in Korean patients with colon cancer (3 out of 147, 2%) through next-generation RNA sequencing (RNA-seq). *NTRK1* fusions were mutually exclusive oncogenic drivers of colon cancer that were accompanied with *in vitro* potential of colony formation and *in vivo* tumorigenicity comparable to KM12, a human colon cancer cell line harboring *TPM3-NTRK1* fusion. *NTRK1*-encoded TrkA protein was prevalent in 11 out of 216 Korean (5.1%) and 28 out of 472 Chinese patients (5.9%) from independent cohorts, respectively. The expression level of TrkA was significantly correlated with *NTRK1* fusion (*p* = 0.0192), which was verified by a fluorescence *in situ* hybridization (FISH). Korean patients with TrkA-positive colon cancer had a marginal but significant shorter overall survival time than TrkA-negative colon cancer [hazard ratio (HR) = 0.5346, 95% confidential interval (CI) = 0.2548-0.9722, *p* = 0.0411]. In addition, KM12 cell line was sensitive to selective TrkA inhibitors. These results demonstrate that *NTRK1* fusion is granted as a clinically relevant target for therapeutic intervention of colon cancer.

## INTRODUCTION

Colorectal cancer (CRC) is the third most commonly diagnosed cancer in the world. The incidence has been rising sharply in some Asian countries, including Japan, Singapore, and Korea, which were previously known as low-risk areas in the last few decades. According to the National Cancer Registry of Korea, the age-standardized incidence rates of total CRC increased by 6.2% and 6.8% for men and women, respectively, between 1999 and 2009 [1]. Although the mortality rate from CRC started to decline among younger generations and women, it is still ranked as one of the most common causes of cancer-related death in Korea [2].

Over the past few decades, significant therapeutic improvements have been made in the treatment of colon cancer. However, most patients with advanced colon cancer are treated with fluoropyrimidine-based chemotherapy in combination with or without irinotecan or oxaliplatin [2]. Recently, molecularly targeted drugs have been developed for use against specific cancers and provide additional clinical benefits to patients. These novel drugs, including cetuximab (Erbitux^®^; Merck KGaA, Germany) and bevacizumab (Avastin^®^; Genentech Inc., USA), have been approved for CRC treatment and improve the survival rate by more than 30 % [3-8]. Nevertheless, there are serious limitations of the targeted drugs to intervene with colon cancer because the molecular characteristics of colon cancer is poorly understood [9, 10], and the clinical benefit of targeted therapeutics is still limited [11, 12].

Recent advances within cancer genomics identify mutually exclusive oncogenic drivers to trigger a personalized treatment in various cancers. However, insufficient clinical characterization of the drivers often led to the poor response in clinical trials with targeted therapeutics [13-15]. Therefore, it is important to associate the long-term clinical outcome of cancer patients with certain genomic alterations through a systemic approach.

The overall goal of this study was to identify clinically confident targets for the therapeutic intervention of colon cancer. To this end, we performed RNA-seq with tumors from Korean patients with colon cancer. From sequence data, we analyzed gene fusions, differential gene expression, and non-synonymous somatic mutations. Gene fusions were verified by the sequencing of the fusion transcripts, FISH, and immunohistochemistry. The oncogenicity of the fusion genes was validated by an *in vitro* colony formation assay and an *in vivo* xenograft study with transformed cell lines with fusion transcripts. The clinical effect of the fusion genes was addressed by analyzing the prevalence and the overall survival of the patients having the genomic alterations from an independent retrospective cohort.

## RESULTS

### The identification of *NTRK1* fusions among Korean patients with colon cancer

**Figure 1:**
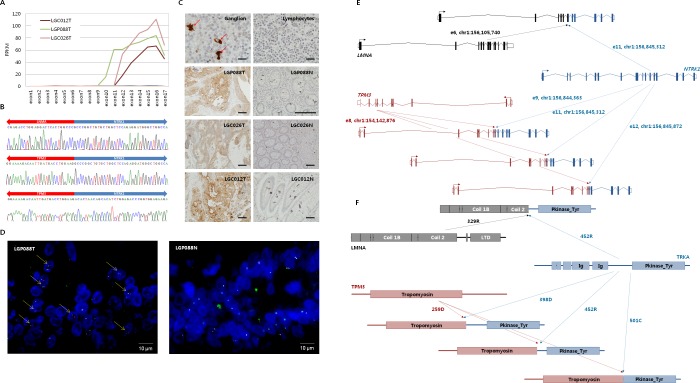
Rearrangement of *NTRK1* in colon cancer of Korean patients **A.** The exon expression of the *NTRK1* gene was exclusively detected in tumors harboring *NTRK1* fusions (Sample ID = LGP088T, LGC026T, LGC012T). **B.** Exon junctions in fusion transcripts of *LMNA-NTRK1* and *TPM3-NTRK1* were confirmed by RT-PCR followed by Sanger sequencing. The *NTRK1* gene at chr1:156,845,312 was rearranged with the *LMNA* gene at chr1:156,105,740, and the *NTRK1* gene at chr1: 156,844,363 or 156,845,312, or 156,845, 872 was rearranged with the *TPM3* gene at chr1:154,142,876, respectively. **C.** Immunohistochemical analysis with anti-TrkA protein, C-terminal antibody showed tumor-specific, cytoplasmic expression of TrkA protein in *NTRK1* fusion-positive samples. Brain ganglions and lymphocytes served as positive and negative control, respectively. The scale bar = 25 μm. **D.** Fluorescence *in situ* hybridization (FISH) assay with split FISH probes to reassure *NTRK1* rearrangement. TexRed-labeled 5′-end *NTRK1* probe is located on chr1:156,390 Kb - 156,814 Kb (red), and FITC-labeled 3′-end *NTRK1* probe on chr1:156,851 Kb - 157,630 Kb (green). Split red and green signals were observed in a representative *NTRK1* fusion-positive tumor tissue (LGP088T), but not in matched normal tissue (LGP088N). Yellow arrows indicate *NTRK1* fusion genes. The scale bar indicates 10 μm. **E.** Schematic rearrangement of *NTRK1* gene. Rearrangement between *LMNA*(e6) and *NTRK1*(e11) generated a single isoform of the fusion transcript, and rearrangement between *TPM3*(e8) and *NTRK1*(e9, e11, e12) generated three isoforms of the fusion transcripts. **F.** Architecture of TrkA fusion proteins. The putative structure of *NTRK1* fusion genes demonstrated that the protein kinase domain of TrkA protein was well conserved.

An RNA-seq cohort was composed of 79 male and 71 female patients with a median age of 60 at diagnosis. The majority of the cancer was located in the ascending colon (48 out of 150; 32%) or in the sigmoid (73 out of 150; 48.7%). Half of the cancers were determined at stage I (12 out of 150; 8%) or stage II (63 out of 150; 42%), and the other half were at stage III (75 out of 150; 50%). Microsatellite analysis revealed that 127 of 150 tumors (84.7%) were microsatellite stable (MSS), and 21 of 150 tumors (14%) had highly unstable microsatellites (MSI-H). Clinical follow-up demonstrated that 23 of 150 patients (15.3%) have experienced disease progression within three years of diagnosis (Table S1). RNA-seq generated a median of 118.5 million mappable reads with a lower base call accuracy of 99% (Q20) = 94.3% (Figure S1 and Table S2). Principle component analysis (PCA) with 18,725 expressed genes from individual tumors revealed three outliers (Figure S2 and Table S2); therefore, 147 tumors and 47 matched normal controls were used for gene fusion analysis. We applied GFP [16], defuse [17], and FusionMap [18], and nine in-frame fusions were found based on two out of three algorithms with discordant paired-end reads, as well as a spanning read cutoff = 10 and a chromosomal distance cutoff = 100 Kb when intrachromosomally rearranged. Gene fusion was validated by exon expression analysis of donor and acceptor genes (Table S3). Those included *PTPRK-RSPO3* in two patients (1.4%), *NAGLU-IKZF3*, *GTF3A-CDK8*, *RAD51AP1-AKAP3*, *RASA1-LOC644100* in each single patient (0.7%), and *LMNA* or *TPM3-NTRK1* in three patients (2%). We then further investigated *NTRK1* fusions because *NTRK1*, which encodes for membrane-bound TrkA protein, has been shown to be rearranged with *TPM3* in colon carcinoma [19] and papillary thyroid carcinoma [20]. *MPRIP-NTRK1* and *CD74-NTRK1* were found in lung adenocarcinoma [21], *TP53-NTRK1* and *LMNA-NTRK1* in Spitzoid neoplasm [22], *RABGAP1L-NTRK1* in intrahepatic cholangiocarcinoma [23], and *NFASC-NTRK1* and *BCAN-NTRK1* in glioblastoma multiforme [24]. To our knowledge, *LMNA-NTRK1* fusion was not reported in colon cancer. Furthermore, the prevalence and the clinical relevance of *NTRK1* fusions remain largely unknown in colon cancer. The exon expression of the *NTRK1* gene was exclusively detected in tumors harboring *NTRK1* fusions (Figure 1A). Subsequently, we confirmed the exon junctions in the fusion transcript of *LMNA-NTRK1* and *TPM3-NTRK1* by Sanger sequencing (Figure 1B), and *NTRK1*-encoded TrkA expression by immunohistochemistry (Figure 1C). *NTRK1* gene fusion was confirmed by a FISH assay with split FISH probes on 5′- and 3′-end of *NTRK1* gene (Figure 1D). Schematic rearrangement of the *NTRK1* gene (Figure 1E) and the architecture of TrkA fusion protein demonstrated that the protein kinase domain of the TrkA protein is well conserved after gene fusion (Figure 1F).

### Mutually exclusive oncogenicity of *NTRK1* fusions in colon cancer

**Figure 2:**
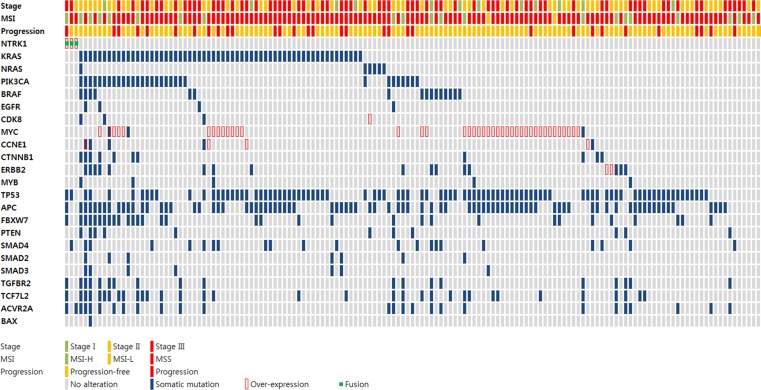
Mutual exclusivity of *NTRK1* fusion to oncogenic mutations in colon cancer The tumor stage was denoted by colored squares (i.e., green for stage I, yellow for stage II, and red for stage III). Microsatellite stability was symbolized as a green square for microsatellite instable-high (MSI-H), a yellow square for microsatellite instable-low (MSI-L), and a red square for microsatellite stable (MSS). Tumor progression was designated by yellow squares (i.e., progression-free) and red (i.e., progression). Any alterations in representative oncogenes and suppressor genes including *NTRK1* in colon cancer were depicted as follows: gray squares for no alteration, blue squares for non-synonymous somatic mutation, red squares for over-expression, and green dots for *NTRK1* fusion. *NTRK1* fusions were mutually exclusive to representative oncogenic mutations in colon cancer. Somatic mutations in various suppressor genes were denoted including *TP53, APC*, and *FBXW7*, particularly in the MSI-H sample.

**Figure 3:**
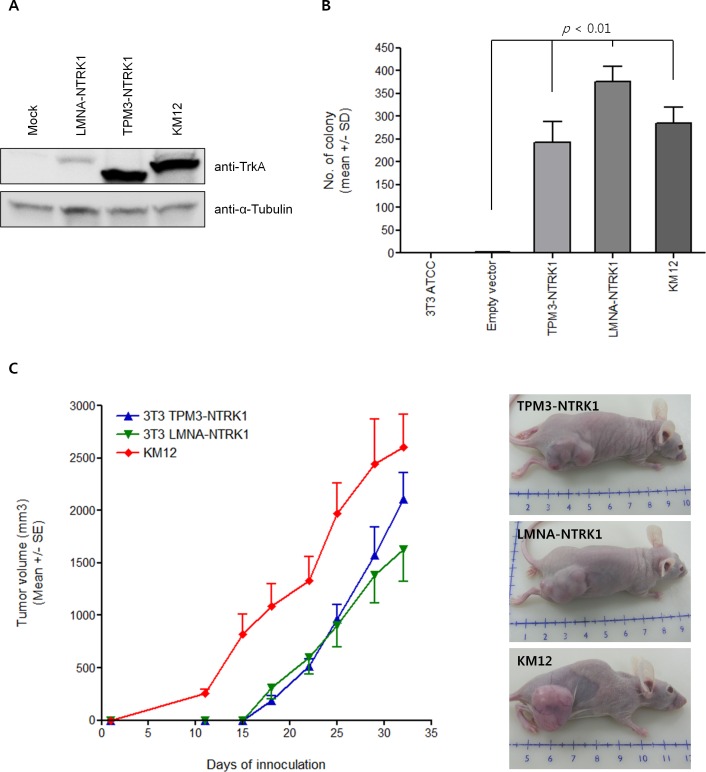
Tumorigenicity assay with transformed NIH3T3 cells **A.** TrkA proteins from a transformed NIH3T3 cell line with *LMNA-NTRK1* or *TPM3-NTRK1* fusion transcript and KM12 were subject to Western blot using the anti-TrkA protein, C-terminal monoclonal antibodies. **B.** A total of 1,200 cells were seeded in the 0.3% of top agarose on the 0.5% of base agarose per well of a 24-well plate and colonies were allowed to form for 3 weeks. NIH3T3 cells transduced with exogenous expression of *LMNA-NTRK1* or *TPM3-NTRK1* formed a small but significantly large number of colonies (376 ± 33 and 243 ± 36 colonies, respectively) in comparison to empty vector control (1 ± 2 colonies, *p* < 0.01), which was comparable to KM12 (285 ± 36 colonies). A colony formation assay was independently performed in triplicate. The number of colonies was denoted as mean ± standard deviation. **C.** NIH3T3 cells transduced with an exogenous expression of *LMNA-NTRK1* or *TPM3-NTRK1* at 1×10^6^ cells per site were inoculated subcutaneously in the right dorsal region of immunocompromised athymic mice (5 mice per group). When the tumor became palpable, the tumor volume was measured every three days until the thirty-second day after inoculation. Transformed NIH3T3 cell-driven tumors were grown comparable to KM12. Tumor volumes were denoted as a mean ± standard error. Representative animals with tumors from *LMNA-NTRK1* or *TPM3-NTRK1* transgene or KM12 were presented. The unit is centimeter.

Whereas the oncogenicity of *NTRK1* fusions was well characterized in papillary thyroid carcinoma [25] and lung adenocarcinoma [21], it remains to be elucidated in colon cancer. To address the oncogenicity of *LMNA-NTRK1* and *TPM3-NTRK1* fusions in colon cancer, we conducted *in silico*, *in vitro*, and *in vivo* approaches. *NTRK1* fusions were mutually exclusive to oncogenic mutations in CRC (Figure 2). Tumors harboring *NTRK1* fusions did not have non-synonymous somatic mutations in *KRAS*, *NRAS*, *PIK3CA* and other putative oncogenes. In contrast, somatic mutations in various suppressor genes, including *TP53*, *APC*, and *FBXW7*, were observed in *NTRK1* fusion-positive tumors. It is noteworthy that most of the suppressor mutations were found in the *NTRK1* fusion-positive tumor with highly unstable microsatellites (Patient ID: LGP088T). We removed potential germline variants, as described in Methods. However, it is likely that unpaired tumor tissues may have more somatic variants than paired tumor tissues. Therefore, the negative selection of somatic variants was preceded and revealed the mutually exclusive oncogenicity of *NTRK1* fusions in colon cancer. Although we performed the clinical follow-up of the patients, most of the patients were diagnosed after 2010 and it is too soon to determine any clinicopathological effects of the *NTRK1* fusions in colon cancer. In subsequent analyses of the oncogenicity of *NTRK1* fusions, we generated *LMNA*(e6)*-NTRK1*(e11) or *TPM3*(e8)*-NTRK1*(e9, e11, e12) fusion transcripts harboring plasmid DNA for cell transformation. TrkA protein from a transformed NIH3T3 cell line with the fusion transcripts was well expressed (Figure 3A). The NIH3T3 cells overexpressing *LMNA-NTRK1* (376 ± 33 colonies) or *TPM3-NTRK1* (243 ± 46 colonies) formed a significantly larger number of colonies than non-transformed cells (1 ± 2 colonies, *p* < 0.01), which was comparable to KM12 (285 ± 36 colonies) (Figure 3B). We then evaluated the tumorigenicity of *NTRK1* fusions by inoculating immunocompromised athymic-mice with the transformed cells. Tumors from the transformed cells were palpable from day 18 of inoculation, and the volumes of the tumors were comparable to KM12 on day 29 of inoculation (Figure 3C). Although the expression level of LMNA-TrkA fusion protein in NIH3T3 cells was less than that of the other two fusion protein-expressing cell lines, oncogenic activity was comparable with that of the other fusion proteins. Therefore, these results demonstrated that *LMNA-NTRK1* and *TPM3-NTRK1* could be mutually exclusive cancer drivers in colon cancer.

### Clinical relevance of *NTRK1* fusions

**Figure 4:**
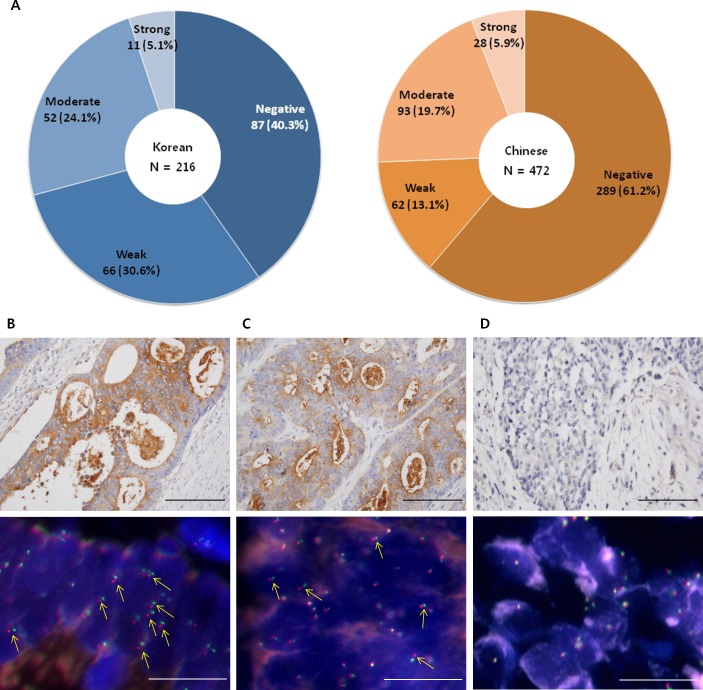
The prevalence of TrkA protein expression in Korean and Chinese patients with colon cancer Pie graphs depict the proportion of subpopulations with a different degree of TrkA protein expression. **A.** In Korean patients, 87 out of 216 patients (40.3%) were TrkA negative, and 66 out of 216 patients (30.6%) had weak TrkA expression (both subpopulations were determined to be TrkA negative). Fifty-two (24.1%) and 11 patients (5.1%) with a moderate to strong expression of TrkA protein were identified and designated as TrkA positive. In Chinese patients, 351 out of 472 patients (74.3%) were TrkA negative, and 121 out of 472 patients (25.6%) were TrkA positive. Out of the Chinese population of 472, 289 (61.2%) and 62 patients (13.1%) were TrkA negative, and 93 (19.7%) and 28 patients (5.9%) were TrkA positive. **B.** Immunohistochemistry represents strong cytoplasmic TrkA expression in a colon cancer (sample ID: Colon_50_FISH01), and FISH analysis confirmed the frequent *NTRK1* rearrangement in the tissue. **C.** Immunohistochemistry represents moderate cytoplasmic TrkA expression in a colon cancer (sample ID: Colon_50_FISH06), and FISH analysis confirmed the less frequent *NTRK1* rearrangement in the tissue. **D.** Immunohistochemistry represents negative cytoplasmic TrkA expression in a colon cancer (sample ID: Colon_50_FISH11), and FISH analysis confirmed that there was no detectable *NTRK1* rearrangement in the tissue. Yellow arrows indicate *NTRK1* fusion genes. The scale bar indicates 10 μm.

**Figure 5:**
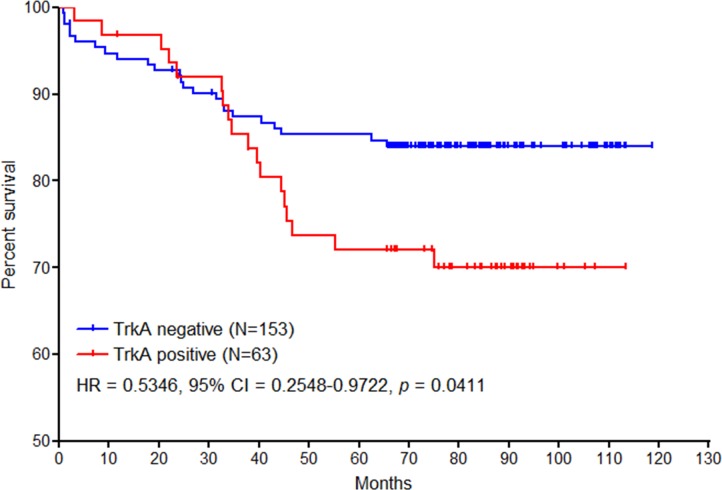
The Kaplan-Meier survival analysis of TrkA-positive and negative patients with colon cancer Out of 216 Korean patients with colon cancer, 153 patients were determined to be TrkA negative, and 63 patients were TrkA positive, respectively. Patient survival was followed up to 118.6 months. The Kaplan-Meier survival analysis demonstrated that the survival time of cytoplasmic TrkA-positive patients was significantly shorter than TrkA-negative patients with T1-3 colon cancer (*p* = 0.0411, HR = 0.5346, 95% CI = 0.2548 to 0.9722).

The clinical relevance of *NTRK1* fusion was assessed with independent cohorts that were comprised of 216 Korean and 472 Chinese patients with colon cancer. We investigated the prevalence of TrkA protein expression by using tissue microarray (TMA) constructed from the cohorts. Immunohistochemical analyses revealed that TrkA was strongly expressed in the cytoplasm of the tumor cells in 11 Korean (5.1%) and 28 Chinese patients (5.9%), respectively, and the frequency of TrkA expression was not significantly different between the two populations (*p* = 0.1657) (Figure 4A). Those frequencies were higher than our expectation from RNA-seq results. To verify the correlation between TrkA protein expression and *NTRK1* fusion, 15 tumor tissues with or without TrkA protein expression from Korean patients were subject to FISH analysis. Split FISH signals were significantly detected in TrkA-positive tumors (*p* = 0.0192, Figure 4C to 4D, Table S4), but not in the TrkA negative tumors (Figure 4E), thus indicating that TrkA protein expression was partly a result of *NTRK1* fusion. Among the 216 Korean patients, 42 patients (24.1%) were had died within 10 years after diagnosis. All of the 216 Korean patients were divided into two groups according to the cytoplasmic TrkA expression level. Clinicopathological characterization revealed that the tumors with cytoplasmic TrkA expression were localized in the left colon (*p* = 0.0504) and frequent at the T3 depth of invasion (*p* = 0.0437). TrkA positive tumors were significantly associated with the occurrence of perineural invasion/lymphovascular emboli (*p* = 0.0429), and most of the tumors were microsatellite stable (*p* = 0.0024) (Table 1). In accordance with the histopathological and molecular status of the tumors, Kaplan-Meier survival analysis demonstrated that the survival time of TrkA-positive patients was marginally but significantly shorter than TrkA-negative patients (Figure 5, HR = 0.5346, 95% CI = 0.2548 to 0.9722, *p* = 0.0411). Taken together, these results imply that *NTRK1* fusion could be a clinically relevant target for the therapeutic intervention of colon cancer.

**Table:** Clinicopathological characteristics of the cohort of 216 Korean patients

	N	Cytoplasmic TrkA		*P* value
		(−)	(+)	
Age (years)	216	70.35	70.22	0.4675
Size (cm)	216	5.199	5.548	0.2959
Sex				0.3351
Male	118	85	33	
Female	98	68	30	
Location				0.0504
Right colon	87	67	20	
Left colon	129	86	43	
Histological type				0.0761
Well	16	13	3	
Moderate	178	120	58	
Poor	10	10	0	
Mucinous	12	10	2	
Invasion depth (T1-2 vs. T3)				0.0437
T1	12	10	2	
T2	23	19	4	
T3	181	124	57	
Perineural invasion (PNI)				0.1315
Negative	149	109	40	
Positive	67	44	23	
Lymphovascular emboli (LVE)				0.2229
Negative	142	103	39	
Positive	74	50	24	
PNI/LVE				0.0429
Negative	119	90	29	
Positive	97	63	34	
Lymphnode metastasis (N0 vs N1-2)				0.015
N0	124	95	29	
N1a	40	23	17	
N1b	32	22	10	
N2a	13	9	4	
N2b	7	4	3	
Microsatellite status				0.0024
MSS	180	119	61	
MSI-L	6	5	1	
MSI-H	30	29	1	

### Therapeutic intervention of *NTRK1* fusion

**Figure 6:**
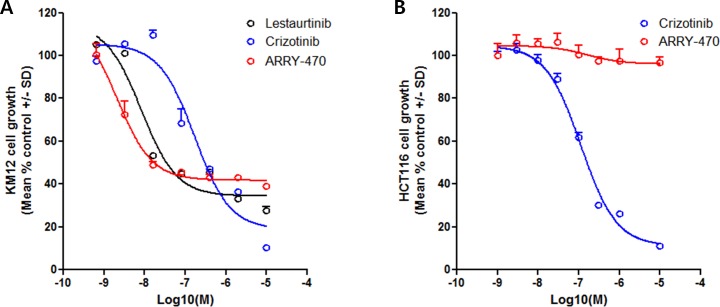
Therapeutic intervention of *NTRK1* fusion-driven cell growth **A.** Therapeutic intervention of *NTRK1* fusion-positive colon cancer cells. Therapeutic intervention of *NTRK1* fusion-driven cell growth was evaluated in KM12 cells with Lestaurtinib, Crizotinib, and ARRY-470. KM12 was treated at 0.64 nM to 10 μM of each compounds for 4 days, and cell growth was evaluated by ATP-Glo Bioluminometric Cell Viability Assay kit (Biotium Inc.). KM12 was sensitive to Lestaurtinib and ARRY-470 with 10.7 nM and 3.2 nM of CC50, respectively. Crizotinib was less potent to inhibit KM12 proliferation with CC50 = 184.8 nM. B. Therapeutic intervention of *NTRK1* fusion-negative colon cancer cells. HCT116 was resistant to ARRY-470. Crizotinib was more potent to inhibit HCT116 proliferation with CC50 = 568 nM.

Therapeutic intervention of *NTRK1* fusion-driven cell growth was evaluated in KM12 cells. Although there are several cell lines harboring *NTRK* fusion, including CUTO-3 lung cancer cells with *MPRIP-NTRK1* fusion and MO-91 acute myeloid leukemia (AML) cells with *ETV6-NTRK3* fusion, KM12 is the only available colon cancer cell line harboring *NTRK1* fusion. Therefore it is useful to use KM12 for high throughput screening of drug candidates for *NTRK1* fusion-positive colon cancer treatment. We determined the 50% cytotoxic concentration (CC50) of Lestaurtinib, Crizotinib and ARRY-470 on KM12. Lestaurtinib is an indolocarbazole derivative to inhibit several tyrosine kinases, including FLT3 and TrkA. It had been in phase II/III trials for the oral treatment of relapsed AML. Crizotinib, a dual inhibitor of hepatocyte growth factor receptor (c-Met/HGFR) kinase and anaplastic lymphoma kinase (ALK), was approved and launched in the U.S. in August 2011 for the treatment of patients with ALK-positive advanced or metastatic non-small cell lung cancer (NSCLC). ARRY-470 (also known as LOXO-101) is a selective TrkA inhibitor in phase I clinical studies for the oral treatment of solid tumors. In 2015, orphan drug designation was assigned to the compound in the U.S. for the treatment of soft tissue sarcoma (https://integrity.thomson-pharma.com). In consistent with the previous study [21], KM12 was sensitive to ARRY-470 (CC50 = 3.2 nM) and Lestaurtinib (CC50 = 10.7 nM). Crizotinib was less potent to inhibit KM12 proliferation with CC50 = 184.8 nM (Figure 6A). This modest activity of Crizotinib could be due to non-TrkA kinase effects. In contrast, ARRY-470 had a poor inhibitory effect on HCT116 which has a mutation in codon 13 of the *RAS* proto-oncogene without *NTRK1* fusion. Crizotinib was more potent to inhibit HCT116 proliferation as expected (Figure 6B). Taken together, these results demonstrated that the use of TrkA kinase-specific inhibitors may provide a new therapeutic strategy for targeted treatment not only for *NTRK1* fusion-driven lung adenocarcinoma and sarcoma but also for colon cancer.

## DISCUSSION

In this study, we applied RNA-seq to identify target candidates for therapeutic intervention of colon cancer. Gene fusion is one of the most important oncogenic mechanisms due to uncontrolled protein expression through the rearrangement of ubiquitously expressed donor genes and acceptor oncogenes. RNA-Seq is advantageous for the discovery of fusion genes compared to the whole genome sequencing because it can identify the fusion genes that are specifically translated into proteins. Therefore, RNA-seq has been widely used to identify active fusion genes. Our results confirm that RNA-seq is an efficient tool to find fusion genes to drive colon cancer. Especially, 3′-*NTRK1* transcripts were expressed only in tumors harboring *LMNA-NTRK1* or *TPM3-NTRK1* fusion genes, and they were precisely concomitant with protein expression (Figure 1). In the previous studies, TCGA did not find *NTRK1* fusion in CRC [9] and Genentech reported a single *TPM3-NTRK1* fusion out of 72 colon tumors (1.4%) [10]. In the current study, we found one *LMNA-NTRK1* and two *TPM3-NTRK1* fusions among 147 Korean patients with colon cancer. The frequency of *NTRK1* fusion was a little higher than in the previous studies [10, 26], which could possibly be explained by high pass paired-end transcriptome sequencing that we applied. In addition, there could be ethnic differences in the frequency of the fusion because we investigated the Korean population only in the current study. In addition to *NTRK1* fusions, we found two *PTPRK-RSPO3* fusions, and each of *NAGLU-IKZF3*, *GTF3A-CDK8*, *RAD51AP1-AKAP3*, and *RASA1-LOC644100* fusion (Table S3). Recurrent *RSPO* fusions have been known to occur in 10% of colon tumors. *RSPO* fusions were mutually exclusive with *APC* mutations and capable of potentiating Wnt signaling [10]. The other fusions have not been reported yet in various tumors, and their oncogenic potential remains to be clarified. Therefore, with the development of sequencing capability and bioinformatics analytics tools, RNA-seq will eventually be an indispensable tool in drug discovery and development.

Most genomic studies on cancer to date use tumor tissues from a prospective collection [9, 10]. Many studies have reported on the clinicopathological characteristics of tumors harboring certain genomic alterations; however, a lack of sufficient follow-up information makes obscure the clinical relevance of certain genomic alterations. Some tumors do not respond to treatments against the genomics-based and preclinically-proven driver oncogenic alterations, and insufficient clinicopathological characterization of the cancer drivers may be the reason for the failure of investigational new targeted drugs in clinical trials. For instance, *RET* fusions were recognized as oncogenic and drug-sensitive rearrangements in approximately 1-2% of lung adenocarcinoma [16, 27] and various multi-kinase inhibitors were actively investigated to treat non-small cell lung cancer. However, the clinical significance of *RET* and/or *RET* fusion genes is not fully understood, which led to the failure to prove the clinical benefit of vandetanib [28], sorafenib [29], and erlotinib [30]. The previous studies support how important the confirmation of the clinicopathological relevance of certain genomic alterations is to provide clinically confident therapeutic targets. Therefore, to obtain clinically relevant therapeutic targets, not only genomics or preclinical evidence of oncogenicity, but also long-term follow-up would be useful. We expect that the current clinical evaluation, accompanied with a genomic analysis of cancer, would accelerate the translational transition of genomic research in cancer and find clinically confident therapeutic targets from cancer genomics.

In this study, we observed that cytoplasmic TrkA was strongly detected in 11 out of 216 Korean patients with colon cancer (5.1%), and it was comparable to the prevalence in Chinese patients (Figure 4). The incidence of cytoplasmic TrkA was higher than that of RNA-seq. We verified the immunohistochemical results with FISH, and it turned out that *NTRK1* fusion was significantly correlated with the expression level of TrkA protein. Therefore, it appears that *NTRK1* fusion could be a possible cause of TrkA overexpression. We cannot exclude the fact that different patient cohorts for RNA-seq and TMA could contribute this discrepancy. Different molecular stability of *NTRK1* mRNA and TrkA protein, and different sensitivity of detection methods by RNA-seq or immunohistochemistry may contribute the discrepancy as well. Cytoplasmic staining in the remainder of the specimen might represent undetected *NTRK1* fusions. Since we applied FISH on tissue slides, probe penetration would be limited compared to cell or metaphase chromosomes. Although RNA-seq is a very sensitive genomic tool to identify fusion gene, it depends on target RNA stability. Therefore, we anticipate that immunohistochemistry accompanied with FISH could be used for the prognosis of colon cancer-harboring *NTRK1* fusion.

TrkA expression was higher in T3 stage tumors than T1 and T2 tumors (*p* = 0.0437). With respect to lymph node metastasis, TrkA expression was elevated in N1 and N2 stage tumors relative to N0 stage tumor (*p* = 0.015). In addition, the frequency of TrkA overexpression was higher in microsatellite-stable tumors than microsatellite-unstable tumors (*p* = 0.0024). In the survival analysis, high TrkA expression was significantly associated with poorer overall survival in colon cancer patients (Figure 5). These results demonstrate that *NTRK1* fusion, as well as *NTRK1* fusion-derived TrkA overexpression, would provide information on the overall survival of the patients with colon cancer. Furthermore, these alterations would give information on the effect of a therapeutic intervention. It suggests that *NTRK1* fusion has the potential prognostic and predictive significance in colon cancer.

Conclusively, we identified *LMNA-NTRK1* and *TPM3-NTRK1* fusion genes in Korean patients with colon cancer through RNA-seq. *NTRK1* fusions were mutually exclusive colon cancer drivers with tumorigenicity in cells and in animals. Clinicopathological analysis demonstrated that the proportion of patients with *NTRK1* fusion-driven TrkA expression was substantial in Korean and Chinese patients with colon cancer, accompanied with poor overall survival. Therefore, *NTRK1* fusion was granted as a therapeutic target to treat colon cancer.

## MATERIALS AND METHODS

### Guideline compliance

All of the methods that are described in this study were carried out in accordance with the approved guidelines for the use of experimental animals and human subjects.

### Patients

This study was approved by the institutional review boards (IRBs) of Chonnam National University Hwasun Hospital and Pusan National University Hospital (PNUH), Korea. Fresh frozen tissues resected between 2008 and 2012 from patients with primary colon cancer and matched normal controls were used in this study. Informed consent was obtained from all patients. Tumor tissues were selected according to the tumor sample inclusion criteria of the International Cancer Genome Consortium (ICGC). Briefly, tumor tissues composed of more than 60% of tumor cells and less than 20% of necrotic cells or normal cells on histological assessment were included. Tumor tissues from patients who had a family history of colon cancer were excluded. In total, 150 tumor and 50 matched normal tissues were analyzed.

### MSI analysis

Tumor DNA and matched normal DNA from formalin-fixed and paraffin-embedded tissues were used for MSI assessment using Bethesda markers (BAT26, D5S346, BAT25, D17S250, D2S123). DNAs were extracted by using the QIAmp DNA FFPE Tissue Kit (Qiagen, Germany). DNA purity and concentration were measured by the ND-1000 spectrophotometer (Nanodrop technology, USA). Multiplex PCR was performed and MSI was determined by amplicon size in consideration of signal intensity. In case of the failure of MSI analysis due to the poor quality of DNA, microsatellite status was predicted based on total number of somatic mutations including DNA polymerase epsilon (*POLE*) alterations.

### RNA-seq

Total RNA was extracted by using the RNAiso Plus (Takara Bio, Japan). Extracted RNA was purified by using the RNeasy Mini Kit accompanied with the DNase I (Qiagen) treatment. RNA integrity was assessed on the Bioanalyzer (Agilent, USA), and tumor RNAs with RNA integrity number (RIN) ≥ 6 were subject to RNA-seq (Table S1). RNA-seq libraries were generated by using the TruSeq RNA sample Preparation Kit (Illumina, USA). Briefly, mRNA was enriched by using poly-T oligo-attached magnetic beads, followed by mRNA fragmentation by acoustic shearing. First-strand cDNA was synthesized by using reverse transcriptase and random hexamers, and second-strand cDNA by using DNA polymerase I and RNase H. cDNA was subject to adapter ligation, and then enriched with PCR to prepare cDNA library. cDNA libraries were sequenced on HiSeq 2000 (Illumina) to obtain around 100 million paired-end reads (2 × 101 bp).

### Sequence data analysis

Sequencing reads from cDNA were aligned with the NCBI human reference genome (hg19) by using GSNAP [31] and TopHat [32] with a 5% mismatch allowance. To minimize mRNA splicing-caused misalignment, sequencing reads were also aligned to a custom human reference cDNA consisting of 161,250 mRNA sequences obtained from public databases (36,742 RefSeq, 73,671 UCSC, and 161,214 Ensembl) [33].

### Gene fusion analysis

In-frame fusion genes were identified by using GFP [16], and cross validated with defuse [17] and FusionMap [18]. In GFP, putative fusion genes were identified by using discordant read pairs on different genes and exon-spanning reads on the exonic fusion breakpoint of chimeric transcripts, followed by serial filtrations in consideration of strand orientation, sequence homology (E-value < 0.01), spurious reads (spanning < 10 bp), and spanning read pattern to remove false positives. deFuse and FusionMap were applied to find the actual location of ambiguously aligned spanning reads with a computed split-read analysis which harbored the fusion breakpoint. The fusion gene was determined when it appears to be at least two different algorithms with discordant paired-end reads and a spanning read cutoff = 10 and a chromosomal distance cutoff = 100 Kb when intrachromosomally rearranged and out-frame fusions were discarded. It follows read depth assessment in each exon after the fusion breakpoint and determines whether they have been abruptly overexpressed [34].

### Differentially expressed gene (DEG) analysis

If not mentioned separately, DEG analysis was performed by using GenePattern at Broad Institute [35]. TopHat alignment was processed using publically available Cufflinks [36] to assemble the reads into transcripts. The number of reads aligned to each gene was normalized by the fragments per kilobase of exon per million (FPKM) [36]. We performed PCA [37] by using the genes with FPKM > 0 in more than 80% of samples and outlier samples that did not adhere to the position of either tumor or normal were excluded in further analyses. It resulted in a total of 18,725 genes from 147 tumor and 47 matched normal tissues. Differential gene expression was computed by a pairwise 2-sided t-test (*p* < 0.05) followed by Benjamini-Hochberg multiple comparison correction [false discovery rate (FDR) < 0.05]. DEGs were determined when the relative expression of genes in tumors was at least 8 times higher than in normal control.

### Non-synonymous somatic mutation analysis

Single nucleotide variances (SNVs) were identified after GSNAP alignment on the custom human reference cDNA. SNVs were defined according to the following criteria: (1) the number of uniquely mapped reads at the position ≥ 2, (2) the average base quality for the position ≥ 20, (3) the allele ratio at the position ≥ 3%, (4) the read depth at the position ≥ 10. Gene annotation was done using RefSeq genes. Potential germline variants were removed by using dbSNP137 at the minor allele frequency > 1% of samples [38], variants in 59 normal Korean individuals [33], and variants in 47 normal tissue counterparts. However, it is likely that unpaired tumor tissues might have more somatic variants than those of paired tumor tissues; therefore, the negative selection of somatic variants proceeded.

### RT-PCR and sanger sequencing

*NTRK1* fusion transcripts were validated with RT-PCR from cDNA by using the following primers and conditions: *LMNA*(e6)-*NTRK1*(e11) fusion transcripts were amplified at 30 cycles of 30 sec at 94°C, 30 sec at 54°C, and 30 sec at 72°C with forward primer 5′-CCA GGT GGA GCA GTA TAA GAA G-3′, reverse primer 5′-TGT GGG TTC TCG ATG ATG TG-3′ for 354-bp product, and *TPM3*(e8)-*NTRK1*(e9, e11, e12) at 30 cycles of 30 sec at 94°C, 30 sec at 55.7°C, and 30 sec at 72°C with forward primer 5′-AAG AAG ATA AAT ATG AGG-3′, reverse primer 5′-CCG TGC CGC ATA TAC TCA AA-3′ for 406, 553, or 712-bp products, respectively. Eluted PCR products were inserted into TOPO TA vector (Life Technologies, USA), and subjected to Sanger sequencing.

### FISH

Commercially available split FISH probes were used to detect *NTRK1* fusion according to the manufacturer's guideline (Abnova, Taiwan). Briefly, a deparaffinized and protease-treated formalin-fixed paraffin-embedded (FFPE) tissue section was denatured at 75°C and then incubated with the TexRed-labeled probe on the 5′ end and the FITC-labeled probe on 3′ end of *NTRK1* overnight. After washing and DAPI counterstaining (Abbott, USA), the number and localization of the hybridization signals were assessed. Tumors were determined to be *NTRK1* fusion positive when more than 15 out of 100 nuclei demonstrated break-apart 5′- and 3′-end signals.

### Immunohistochemistry and clinicopathological characterization

TrkA expression was confirmed on archival FFPE tumor tissues and matched normal tissues with anti-TrkA protein, C-terminal monoclonal antibody (OriGene, USA). Brain ganglions and lymphocytes served as positive and negative control, respectively. The prevalence of TrkA protein expression was evaluated by using TMA constructed from 216 Korean patients (PNUH cohort) and 472 Chinese patients with colon cancer (Biomax, USA). HE-stained sections from each block were made to define representative tumor regions. TMAs were comprised of two cores of 2 mm each, and TMA blocks were used for immunostaining. TrkA immunostaining was scored semiquantitatively as follows: negative (−), weak (+), moderate (++), strong (+++). Moderate-to-strong signal was determined to be TrkA positive.

### Exogenous expression of the *NTRK1* fusion gene in NIH3T3 cells

Plasmids containing genes of interest were purchased from Origene (USA). *LMNA*(e6)-*NTRK1*(e11) and *TPM3*(e8)-*NTRK1*(e9, e11, e12) fusion transcripts were generated by the Overlap Extension PCR. Once PCR products containing the fusion transcripts were generated, they were cloned into the pcDNA3.1 (Life Technologies, USA) harboring internal ribosome entry site (IRES)-*GFP* gene using the Cold Fusion Cloning Kit (System Biosciences, USA), and confirmed by Sanger sequencing. NIH3T3 cells were purchased from the Korean Cell Line Bank (KCLB, Korea) and maintained in glutamine-containing DMEM medium supplemented with 10% heat inactivated New Born Calf Serum, penicillin and streptomycin. All reagents were purchased from Life Technologies if not mentioned separately. The fusion gene expression vector or empty vector was transduced into the NIH3T3 cells by using the FUGENE 6 (Roche, Germany). Cells were selected for at least 20 days by using 800 μg/mL of G418 after transduction, and GFP expressing cells were sorted by BD FACSAriaTM II (BD Biosciences, USA). The exogenous protein expression was tested by Western blotting. Cells were lysed with the RIPA buffer (50 mM Tris pH 7.5, 150 mM NaCl, 1 % NP-40, 0.1 % SDS, 50 mM NaF, 1 mM NaVO_4_, 1 mM EDTA, 1 % Sodium deoxycholate) and applied to the SDS-PAGE. Anti-TrkA antibody was purchased from Abbiotech (USA) and anti-α-tubulin was obtained from Cell Signaling Technologies (USA).

### Colony formation and xenograft assay

The clonogenicity and tumorigenicity of NIH3T3 cells with and exogenous expression of *LMNA*(e6)-*NTRK1*(e11) or *TPM3*(e8)-*NTRK1*(e9) was evaluated. A total of 1,200 cells were seeded in 0.3% of top agarose on 0.5% of base agarose per well of a 24-well plate, and colonies were allowed to form for 3 weeks. NIH3T3 cells transduced with empty vector and KM12 (KCLB) were used for negative and positive controls, respectively. Colonies were stained with 0.05% crystal violet and counted under stereomicroscopy. A colony formation assay was independently performed in triplicate. An *in vivo* tumorigenicity study was approved by the Institutional Animal Care and Use Committee (IACUC) at LG Life Sciences, R&D Park, Korea. Immunocompromised athymic female Balb/c mice were purchased from Oriental Bio (Korea) at 6 weeks of age. After 1-week of acclimation, the animals were randomly allocated into groups based on bodyweight and health condition. Transformed NIH3T3 cells or KM12 cells at 1×10^6^ cells per site were inoculated subcutaneously in the right dorsal region of immunocompromised nude mice (5 mice per group). When the tumor became palpable, the tumor volume was measured every three days until day 32 after inoculation.

### Drug screening

Lestaurtinib and Crizotinib were purchased from Tocris Bioscience (Bristol, UK). ARRY-470 was supplied by LG Life Sciences. KM12 and HCT116 (KCLB) were treated at 0.64 nM to 10 μM of each compounds for 4 days, and cell growth was evaluated by the ATP-Glo Bioluminometric Cell Viability Assay Kit (Biotium Inc., USA).

### Statistical analyses

Parametric data were tested for equal variance by applying Bartlett's test. When not significant, data were subject to one-way ANOVA followed by post-hoc Dunnett's multiple comparison test (*p* < 0.05). Categorical data were subject to a chi-square test (*p* < 0.05).

## SUPPLEMENTARY MATERIAL FIGURES AND TABLES


