# Supplementary material for: NTRK1 fusions for the therapeutic intervention of Korean patients with colon cancer
Source: Oncotarget. 2015 Dec 22;7(7):8399–412. doi: 10.18632/oncotarget.6724 (PMC4885001; doi:10.18632/oncotarget.6724)
Supplement: Supplementary file 1 [file oncotarget-07-8399-s001.pdf]

NTRK1 fusions for the therapeutic intervention of Korean patients with colon cancer

Supplementary Material

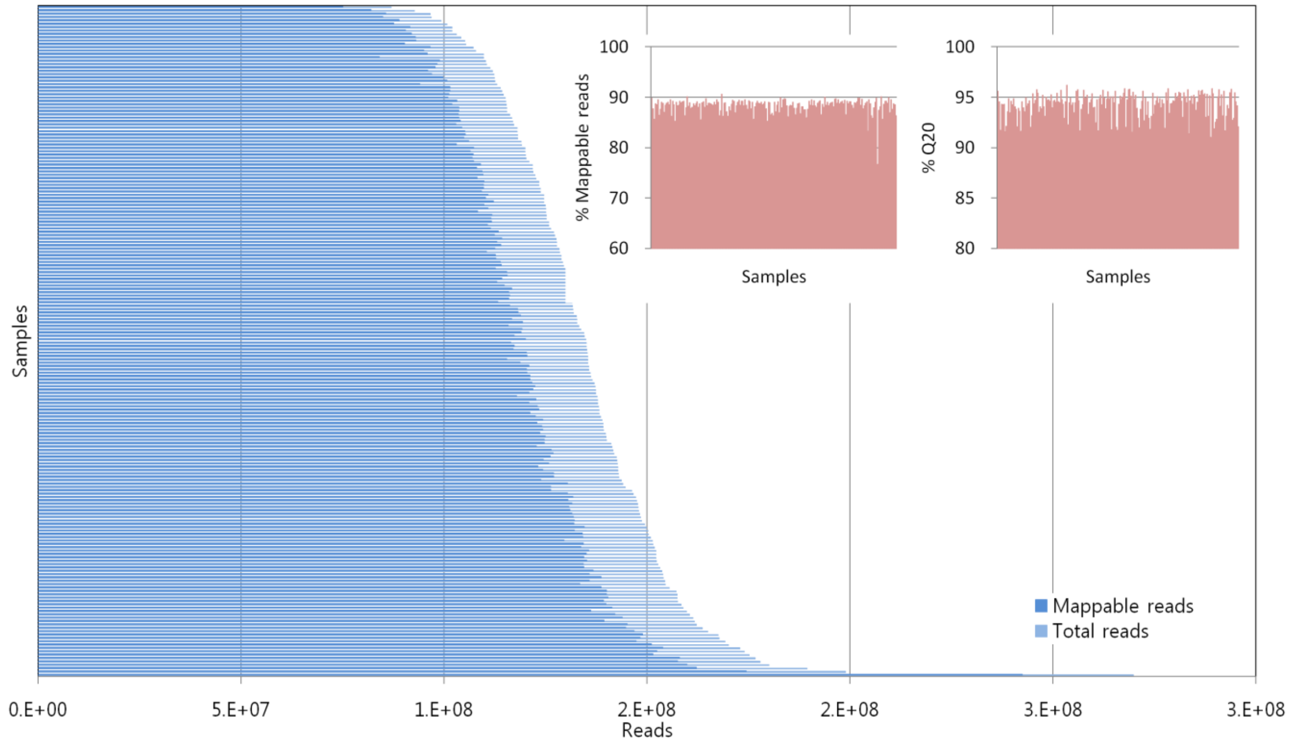

Figure S1. Throughput of next-generation RNA sequencing (RNA-seq). RNA-seq through HiSeq 2000 generated a median of 135 million (M) pair-end total reads (2 x 101 bp, min = 87M, max = 269.9M) and median 118.5M paired-end mappable reads (min = 75.1M, max = 242.4M). The x-axis indicates the number of pair-end reads and the y-axis denotes individual samples. Dark-blue bars indicate mappable reads and pale blue bars denote total reads. The left insert depicts the percentage of mappable reads of individual samples with a median = 88.7% (min = 76.7%, max = 90.6%). The right insert depicts the accuracy of the base call. The median Q20 is 94.6% (min = 91%, max = 96.2%).

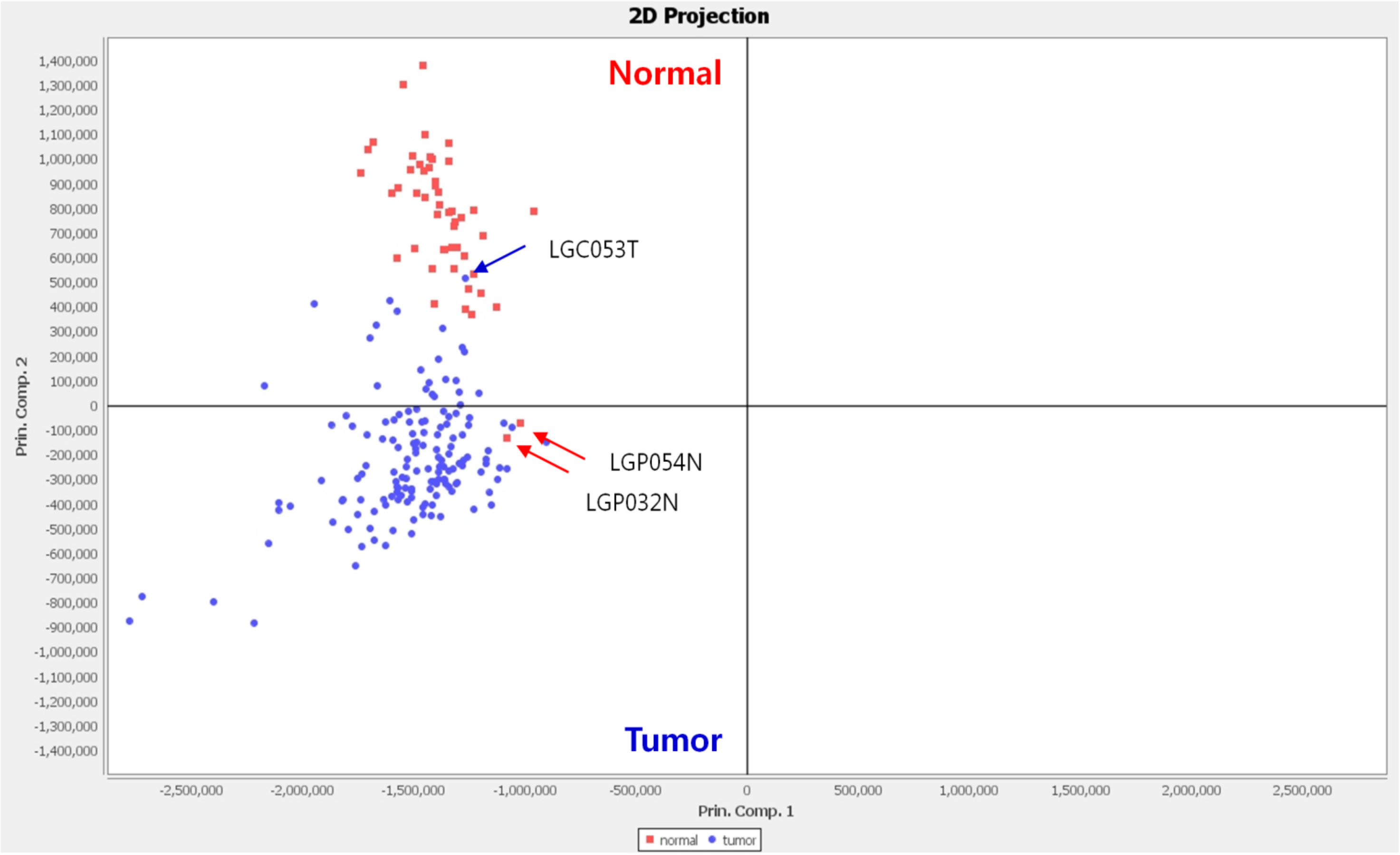

Figure S2. Principle Component Analysis (PCA). The gene set for PCA analysis was selected in consideration of the gene expression and sample coverage (refer to Methods). PCA analysis with 18,725 expressed genes of individual tumor and matched normal tissues revealed three outliers including one tumor tissue (Patient ID = LGC053T) and two normal tissues (LGP054N, LGP032N). These tissues and matched tissues were excluded in further analysis. Red dots indicate normal samples, blue dots signify tumors. Blue and red arrows indicate outliers.

Table S1. Clinicopathological information of Korean patients with colon cancer for RNA-seq

Patient ID	Location	Gender	Age	Histology	Tumor content (%)	Stage	T	N	M	Microsatellite status	Diagnosed year	Normal matched (Pair=1)	Progress in 3-year (Event=1)
LGC001T	SIGMOID	F	60	ADENOCARCINOMA	60	II	3	0	0	MSS	2011	1	
LGC002T	SIGMOID	M	64	ADENOCARCINOMA	80	III	3	1a	0	MSS	2011	1	
LGC003T	ASCENDING	F	62	ADENOCARCINOMA	80	II	3	0	0	MSI-H	2011	1	
LGC004T	ASCENDING	F	30	ADENOCARCINOMA	80	II	3	0	0	MSI-H^	2011		1
LGC005T	ASCENDING	F	75	ADENOCARCINOMA	70	III	3	2b	0	MSI-H	2011		
LGC006T	TRANSVERSE	M	73	ADENOCARCINOMA	90	III	3	1a	0	MSI-H	2011		
LGC007T	ASCENDING	F	41	ADENOCARCINOMA	80	III	3	1	0	MSI-H	2011		
LGC008T	SIGMOID	F	63	ADENOCARCINOMA	80	III	3	1b	0	MSS	2011	1	
LGC009T	SIGMOID	M	52	ADENOCARCINOMA	60	II	3	0	0	MSI-H	2011		1
LGC010T	ASCENDING	F	67	ADENOCARCINOMA	80	II	4a	0	0	MSS	2011	1	1
LGC011T	TRANSVERSE	F	52	ADENOCARCINOMA	70	III	3	2	0	MSS	2010		
LGC012T	ASCENDING	F	78	ADENOCARCINOMA	80	III	4a	2a	0	MSS	2011		
LGC013T	DESCENDING	M	42	ADENOCARCINOMA	70	II	3	0	0	MSS	2011		
LGC014T	SIGMOID	M	42	ADENOCARCINOMA	70	III	3	1a	0	MSS	2011		
LGC015T	ASCENDING	M	64	ADENOCARCINOMA	70	II	3	0	0	MSS^	2010	1	
LGC016T	ASCENDING	F	70	ADENOCARCINOMA	95	III	3	2a	0	MSI-H	2011		
LGC017T	SIGMOID	F	60	ADENOCARCINOMA	60	III	3	1	0	MSS	2010	1	1
LGC018T	ASCENDING	F	57	ADENOCARCINOMA	60	III	3	2b	0	MSS	2011		1

LGC019T	ASCENDING	F	56	ADENOCARCINOMA	70	III	3	2	0	MSS	2010		1
LGC020T	ASCENDING	F	64	ADENOCARCINOMA	70	II	3	0	0	MSS	2011	1	
LGC021T	ASCENDING	F	79	ADENOCARCINOMA	60	III	3	1	0	MSS	2010		1
LGC022T	DESCENDING	F	62	ADENOCARCINOMA	70	II	3	0	0	MSS	2010	1	
LGC023T	SIGMOID	F	60	ADENOCARCINOMA	80	III	3	1a	0	MSS	2011	1	
LGC024T	SIGMOID	F	62	ADENOCARCINOMA	70	III	3	1	0	MSS	2010	1	
LGC025T	TRANSVERSE	F	58	ADENOCARCINOMA	60	II	3	0	0	MSS	2010		
LGC026T	SIGMOID	M	65	ADENOCARCINOMA	70	II	3	0	0	MSS^	2010	1	
LGC027T	SIGMOID	M	68	ADENOCARCINOMA	70	II	3	0	0	MSS^	2010	1	
LGC028T	HEPATIC	M	65	ADENOCARCINOMA	70	II	3	0	0	MSS	2010	1	
LGC029T	RECTOSIGMOID	M	59	ADENOCARCINOMA	60	II	3	0	0	MSS^	2010		
LGC030T	SIGMOID	M	62	ADENOCARCINOMA	60	III	4a	2b	0	MSS	2011	1	
LGC031T	ASCENDING	M	68	ADENOCARCINOMA	70	III	3	1	0	MSS	2010	1	
LGC032T	SIGMOID	M	70	MICROPAPILLARY ADENOCARCINOMA	80	III	3	2b	0	MSS^	2011		
LGC033T	SIGMOID	M	62	ADENOCARCINOMA	70	II	3	0	0	MSS	2010	1	
LGC034T	SIGMOID	F	80	ADENOCARCINOMA	70	III	3	1	0	MSS	2010		
LGC035T	ASCENDING	F	75	ADENOCARCINOMA	90	III	4a	2a	0	MSS	2011		1
LGC036T	SIGMOID	M	52	ADENOCARCINOMA	80	II	2	0	0	MSS	2010		
LGC037T	SIGMOID	F	68	ADENOCARCINOMA	60	II	3	0	0	MSS	2010	1	
LGC038T	SIGMOID	M	59	ADENOCARCINOMA	70	II	3	0	0	MSS	2011		
LGC039T	ASCENDING	M	51	ADENOCARCINOMA	80	II	3	0	0	MSI-H^	2010		
LGC040T	PROXIMAL SIGMOID	F	71	ADENOCARCINOMA	90	III	3	2b	0	MSS	2011		
LGC041T	SIGMOID	M	76	ADENOCARCINOMA	80	III	4	2	0	MSS	2010		
LGC042	TRANSVERS	F	70	ADENOCARCINOMA	80	III	3	1	0	MSI-H	2010		

T	E												
LGC043T	RECTOSIGMOID	M	68	ADENOCARCINOMA	60	II	2	0	0	MSS^	2010	1	
LGC044T	ASCENDING	F	60	ADENOCARCINOMA	60	II	3	0	0	MSI-H	2011		
LGC045T	ASCENDING	M	50	ADENOCARCINOMA	60	II	2	0	0	MSI-H	2010		
LGC046T	ASCENDING	M	55	ADENOCARCINOMA	70	III	3	1b	0 or 1	MSS	2010		1
LGC047T	ASCENDING	M	78	ADENOCARCINOMA	60	III	3	2a	0	MSI-H	2010		
LGC048T	ASCENDING	M	56	ADENOCARCINOMA	70	II	3	0	0	MSS	2010		
LGC049T	SIGMOID	M	63	ADENOCARCINOMA	70	II	3	0	0	MSI-L	2010	1	
LGC050T	ASCENDING	M	61	ADENOCARCINOMA	70	II	3	0	0	MSS	2010	1	
LGC051T	ASCENDING	M	69	ADENOCARCINOMA, MODERATELY DIFFERENTIATED	60	II	3	0	0	MSS	2012	1	
LGC052T	RECTOSIGMOID	F	71	ADENOCARCINOMA	70	III	3	1b	0	MSS	2010		1
LGC053T	ASCENDING	F	64	ADENOCARCINOMA	70	III	3	1b	0	MSS	2012	1	
LGC054T	TRANSVERSE	M	74	ADENOCARCINOMA, MODERATELY DIFFERENTIATED	60	III	4a	2b	0	MSS^	2012		1
LGC055T	SIGMOID	M	65	ADENOCARCINOMA, MODERATELY DIFFERENTIATED	70	II	4a	0	0	MSS	2012	1	
LGC056T	ASCENDING	F	64	ADENOCARCINOMA, MODERATELY DIFFERENTIATED	70	II	3	0	0	MSS	2012	1	
LGC057T	DESCENDING	M	46	ADENOCARCINOMA, MODERATELY DIFFERENTIATED	70	II	3	1a	0	MSS^	2012		
LGC058T	SIGMOID	M	61	ADENOCARCINOMA, MODERATELY DIFFERENTIATED	70	II	3	1a	0	MSS	2012	1	
LGC059T	DESCENDING	M	52	ADENOCARCINOMA, MODERATELY DIFFERENTIATED	80	II	3	0	0	MSS	2012		
LGC060T	SIGMOID	M	58	ADENOCARCINOMA, MODERATELY DIFFERENTIATED	80	II	3	1a	0	MSS	2012		1
LGC061T	ASCENDING	M	55	ADENOCARCINOMA	80	III	4a	1b	0	MSS^	2010		
LGC062T	RECTOSIGMOID	F	59	ADENOCARCINOMA	80	II	3	0	0	MSS^	2010		1
LGP001T	SIGMOID	M	71	ADENOCARCINOMA	70	III	3	1a	0	MSS	2010		
LGP002T	CECUM	F	48	ADENOCARCINOMA	90	II	3	0	0	MSI-H	2010		
LGP003T	SIGMOID	F	61	ADENOCARCINOMA	80	III	3	2b	0	MSS	2010		

LGP004T	ASCENDING	F	41	ADENOCARCINOMA	90	II	3	0	0	MSI-H	2010		
LGP005T	SIGMOID	F	74	ADENOCARCINOMA	90	III	3	1b	0	MSS	2010		
LGP006T	ASCENDING	M	79	ADENOCARCINOMA	95	III	4a	1b	0	MSS	2010		1
LGP007T	ASCENDING	F	77	ADENOCARCINOMA	90	III	3	2b	0	MSS	2010		
LGP008T	ASCENDING	M	56	ADENOCARCINOMA	80	III	4a	1b	0	MSS	2010		
LGP009T	SIGMOID	F	53	ADENOCARCINOMA	80	III	4a	2b	0	MSS	2010	1	
LGP010T	SIGMOID	M	50	ADENOCARCINOMA	70	III	3	2a	0	MSS	2010		
LGP011T	SIGMOID	M	68	ADENOCARCINOMA	90	III	3	2b	0	MSS	2010		
LGP012T	SIGMOID	M	68	ADENOCARCINOMA	60	III	3	1a	0	MSS	2010		
LGP013T	SIGMOID	M	49	ADENOCARCINOMA	90	II	3	0	0	MSS	2010		1
LGP014T	ASCENDING	M	61	ADENOCARCINOMA	70	III	4a	1b	0	MSS	2010		
LGP015T	SIGMOID	F	60	ADENOCARCINOMA	70	III	2	1a	0	MSS	2010		
LGP016T	ASCENDING	F	58	ADENOCARCINOMA	90	III	3	1b	0	MSS	2010	1	
LGP017T	ASCENDING	F	75	ADENOCARCINOMA	70	III	3	2a	0	MSS	2010	1	1
LGP018T	ASCENDING	F	55	ADENOCARCINOMA	60	II	3	0	0	MSI-H	2010		
LGP019T	SIGMOID	M	56	ADENOCARCINOMA	60	III	4a	2a	0	MSS	2010		
LGP020T	SIGMOID	M	58	ADENOCARCINOMA	90	II	3	0	0	MSS	2010	1	
LGP021T	SIGMOID	M	30	ADENOCARCINOMA	95	III	3	1b	0	MSS	2010		
LGP022T	SIGMOID	F	59	ADENOCARCINOMA	90	III	3	1a	0	MSS	2010		
LGP023T	SIGMOID	F	44	ADENOCARCINOMA	80	II	3	0	0	MSS	2010		
LGP024T	SIGMOID	M	53	ADENOCARCINOMA	60	II	3	0	0	MSS	2010		
LGP025T	DESCENDING	F	51	ADENOCARCINOMA	90	II	3	0	0	MSS	2010	1	
LGP026T	SIGMOID	F	52	ADENOCARCINOMA	80	II	3	0	0	MSS	2010		
LGP027	ASCENDING	F	43	ADENOCARCINOMA, WELL	90	I	2	0	0	MSI-H	2011		

T				DIFFERENTIATED									
LGP028T	SIGMOID	F	68	ADENOCARCINOMA, MODERATELY DIFFERENTIATED	90	III	4a	1a	0	MSS	2011		
LGP029T	SIGMOID	F	61	ADENOCARCINOMA, MODERATELY DIFFERENTIATED	80	I	2	0	0	MSS	2011	1	
LGP030T	ASCENDING	M	58	ADENOCARCINOMA, MODERATELY DIFFERENTIATED	80	III	4a	1b	0	MSS	2011		
LGP031T	DESCENDING	M	59	ADENOCARCINOMA, WELL DIFFERENTIATED	80	II	3	0	0	MSS	2011	1	
LGP032T	SIGMOID	M	85	ADENOCARCINOMA, MODERATELY DIFFERENTIATED	80	III	3	1a	0	MSS	2011	1	
LGP033T	SIGMOID	F	42	ADENOCARCINOMA, MODERATELY DIFFERENTIATED	60	III	3	2a	0	MSS	2011	1	
LGP034T	SIGMOID	F	41	ADENOCARCINOMA, MODERATELY DIFFERENTIATED	70	III	4a	2b	0	MSS	2011	1	
LGP035T	DESCENDING	F	47	ADENOCARCINOMA	80	III	3	1b	0	MSS	2009		
LGP036T	ASCENDING	F	48	ADENOCARCINOMA, MODERATELY DIFFERENTIATED	75	II	3	0	0	MSS	2008		
LGP037T	SIGMOID	M	28	ADENOCARCINOMA, MODERATELY DIFFERENTIATED	80	II	3	0	0	MSS	2008	1	1
LGP038T	SIGMOID	F	52	ADENOCARCINOMA	90	II	3	0	0	MSS	2009		
LGP039T	SIGMOID	F	67	ADENOCARCINOMA	80	III	3	1a	0	MSS	2009		
LGP040T	SIGMOID	M	73	ADENOCARCINOMA	90	III	3	1a	0	MSS	2009		1
LGP041T	ASCENDING	F	74	ADENOCARCINOMA	60	III	4a	1a	0	MSS	2009		1
LGP042T	SIGMOID	F	73	ADENOCARCINOMA	80	III	3	1a	0	MSS	2009		1
LGP043T	ASCENDING	M	75	ADENOCARCINOMA	60	III	4a	1a	0	MSS	2009		
LGP044T	SIGMOID	M	60	ADENOCARCINOMA, MODERATELY DIFFERENTIATED	60	II	3	0	0	MSS	2011		
LGP045T	SIGMOID	F	29	ADENOCARCINOMA, MODERATELY DIFFERENTIATED	70	I	2	0	0	MSS	2011		
LGP046T	DESCENDING	M	55	ADENOCARCINOMA, MODERATELY DIFFERENTIATED	60	II	3	0	0	MSS	2011		
LGP047T	SIGMOID	M	60	ADENOCARCINOMA, MODERATELY DIFFERENTIATED	100	II	3	0	0	MSS	2011		
LGP048T	SIGMOID	M	75	ADENOCARCINOMA	90	III	3	2a	0	MSS	2012		
LGP049T	DESCENDING	M	59	ADENOCARCINOMA	70	II	3	0	0	MSS	2012		
LGP050T	ASCENDING	M	57	ADENOCARCINOMA	90	II	3	0	0	MSI-H	2012		1

LGP051 T	SIGMOID	M	57	ADENOCARCINOMA	100	II	3	0	0	MSS	2012		
LGP052 T	SIGMOID	F	79	ADENOCARCINOMA	90	III	3	1b	0	MSS	2012	1	
LGP053 T	SIGMOID	M	47	ADENOCARCINOMA	95	II	3	0	0	MSS	2012		
LGP054 T	SIGMOID	M	68	ADENOCARCINOMA	60	III	3	1b	0	MSS	2012	1	
LGP055 T	ASCENDING	F	57	ADENOCARCINOMA	80	I	2	0	0	MSI-H	2012		
LGP056 T	DESCENDIN G	M	65	ADENOCARCINOMA	60	III	3	2a	0	MSS	2012	1	
LGP057 T	ASCENDING	M	52	ADENOCARCINOMA	100	II	3	0	0	MSS	2012	1	
LGP058 T	RECTOSIGM OID	F	50	ADENOCARCINOMA	80	III	4b	1b	0	MSS	2012	1	
LGP059 T	SIGMOID	M	88	ADENOCARCINOMA	100	III	3	1a	0	MSI-L	2012	1	
LGP060 T	SIGMOID	F	64	ADENOCARCINOMA	95	I	2	0	0	MSS	2012	1	
LGP061 T	SIGMOID	M	56	ADENOCARCINOMA	60	II	3	0	0	MSS	2012	1	
LGP062 T	SIGMOID	F	78	ADENOCARCINOMA	100	III	3	1a	0	MSS	2012	1	
LGP063 T	SIGMOID	M	69	ADENOCARCINOMA	85	I	2	0	0	MSS	2012	1	
LGP064 T	DESCENDIN G	M	47	ADENOCARCINOMA	100	II	3	0	0	MSI-H	2012	1	
LGP065 T	SIGMOID	M	57	ADENOCARCINOMA	80	II	3	0	0	MSS	2012	1	1
LGP066 T	SIGMOID	M	74	ADENOCARCINOMA	60	III	4a	2a	0	MSS	2012	1	
LGP067 T	SIGMOID	M	61	ADENOCARCINOMA	95	III	3	1b	0	MSS	2012	1	
LGP068 T	ASCENDING	M	53	ADENOCARCINOMA	70	II	3	0	0	MSS	2012	1	
LGP069 T	TRANSVERS E	M	78	ADENOCARCINOMA	90	III	3	1b	0	MSS	2012	1	
LGP070 T	ASCENDING	F	51	ADENOCARCINOMA	60	I	2	0	0	MSS	2012	1	
LGP071 T	SIGMOID	M	50	ADENOCARCINOMA	90	I	2	0	0	MSS	2012	1	
LGP072 T	ASCENDING	F	53	ADENOCARCINOMA	80	II	3	0	0	MSS	2012	1	
LGP073 T	RECTOSIGM OID	M	42	ADENOCARCINOMA	70	II	3	0	0	MSS	2012		
LGP074	SIGMOID	F	72	ADENOCARCINOMA	90	III	3	1a	0	MSS	2012	1	

T													
LGP075 T	CECUM	M	63	ADENOCARCINOMA	80	I	2	0	0	MSS	2012	1	
LGP076 T	ASCENDING	F	51	ADENOCARCINOMA	80	II	3	0	0	MSI-H	2009		
LGP077 T	SIGMOID	F	60	ADENOCARCINOMA	80	II	3	0	0	MSS	2009		
LGP078 T	SIGMOID	M	59	ADENOCARCINOMA	100	III	3	1b	0	MSS	2009		
LGP079 T	ASCENDING	M	57	ADENOCARCINOMA, MODERATELY DIFFERENTIATED	80	III	2	1a	0	MSS	2011	1	
LGP080 T	SIGMOID	F	43	ADENOCARCINOMA, MODERATELY DIFFERENTIATED	70	II	3	0	0	MSS	2011		
LGP081 T	RECTOSIGMOID	F	69	ADENOCARCINOMA, POORLY DIFFERENTIATED	60	III	3	1b	0	MSS	2011	1	
LGP082 T	SIGMOID	M	72	ADENOCARCINOMA, MODERATELY DIFFERENTIATED	80	III	3	1a	0	MSS	2011	1	
LGP083 T	SIGMOID	F	69	ADENOCARCINOMA, MODERATELY DIFFERENTIATED	90	I	2	0	0	MSS	2011	1	
LGP084 T	SIGMOID	M	48	ADENOCARCINOMA	60	I	2	0	0	MSS	2009		
LGP085 T	SIGMOID	M	78	ADENOCARCINOMA	90	III	4a	2a	0	MSS	2009		
LGP086 T	DESCENDING	F	59	ADENOCARCINOMA	80	I	1	0	0	MSS	2010		
LGP087 T	ASCENDING	F	72	ADENOCARCINOMA	90	III	3	2a	0	MSS	2010		
LGP088 T	ASCENDING	F	71	ADENOCARCINOMA	90	III	3	1a	0	MSI-H	2010		1

^Microsatellite status was predicted in consideration of the total number of somatic mutations and the presence of the mutations in DNA polymerase epsilon (POLE) gene

Table S2. Summary of RNA-seq quality control

Patient ID	RIN[1]	Total bases	Mappable bases	%Mappable bases	Total reads	Mappable reads	%Mappable reads	%Q20[2]	PCA
LGC001N	9.3	1.21E+10	1.08E+10	89	1.20E+08	1.07E+08	89	96	
LGC001T	9.7	1.32E+10	1.17E+10	88	1.31E+08	1.16E+08	88	95	
LGC002N	7.2	1.09E+10	9.60E+09	88	1.08E+08	9.50E+07	88	93	
LGC002T	9.9	1.16E+10	1.02E+10	88	1.15E+08	1.01E+08	88	93	
LGC003N	9.9	1.36E+10	1.18E+10	87	1.35E+08	1.17E+08	87	96	
LGC003T	9.7	1.31E+10	1.15E+10	88	1.29E+08	1.14E+08	88	95	
LGC004T	10.0	1.43E+10	1.28E+10	89	1.42E+08	1.27E+08	89	96	
LGC005T	9.3	1.60E+10	1.41E+10	88	1.58E+08	1.40E+08	88	94	
LGC006T	9.7	1.91E+10	1.64E+10	86	1.89E+08	1.62E+08	86	92	
LGC007T	10.0	1.22E+10	1.08E+10	89	1.21E+08	1.07E+08	89	93	
LGC008N	7.5	1.54E+10	1.36E+10	89	1.52E+08	1.35E+08	89	93	
LGC008T	7.0	1.71E+10	1.49E+10	87	1.69E+08	1.47E+08	87	93	
LGC009T	9.6	1.03E+10	9.25E+09	90	1.02E+08	9.16E+07	90	96	
LGC010N	8.8	1.17E+10	1.05E+10	90	1.15E+08	1.04E+08	90	96	
LGC010T	10.0	1.24E+10	1.09E+10	88	1.23E+08	1.08E+08	88	95	
LGC011T	8.5	1.00E+10	8.98E+09	90	9.93E+07	8.89E+07	90	93	
LGC012T	9.8	1.59E+10	1.41E+10	88	1.58E+08	1.39E+08	88	94	
LGC013T	9.3	1.34E+10	1.21E+10	90	1.33E+08	1.19E+08	90	96	
LGC014T	10.0	1.75E+10	1.55E+10	89	1.73E+08	1.54E+08	89	94	
LGC015N	8.3	1.44E+10	1.27E+10	89	1.42E+08	1.26E+08	89	96	
LGC015T	9.5	1.14E+10	1.01E+10	89	1.12E+08	9.97E+07	89	95	
LGC016T	10.0	1.39E+10	1.22E+10	88	1.38E+08	1.21E+08	88	92	
LGC017N	8.9	1.30E+10	1.15E+10	88	1.29E+08	1.14E+08	88	93	
LGC017T	7.1	1.26E+10	1.11E+10	88	1.25E+08	1.10E+08	88	93	
LGC018T	9.4	1.29E+10	1.14E+10	88	1.28E+08	1.13E+08	88	93	
LGC019T	9.9	1.54E+10	1.36E+10	89	1.52E+08	1.35E+08	89	94	
LGC020N	8.7	1.08E+10	9.76E+09	90	1.07E+08	9.66E+07	90	96	
LGC020T	9.6	1.33E+10	1.17E+10	88	1.31E+08	1.16E+08	88	96	
LGC021T	7.8	1.26E+10	1.13E+10	89	1.25E+08	1.12E+08	89	94	
LGC022N	9.9	1.26E+10	1.13E+10	90	1.25E+08	1.12E+08	90	96	

LGC022T	8.2	1.11E+10	9.69E+09	87	1.10E+08	9.59E+07	87	95	
LGC023N	9.1	1.23E+10	1.10E+10	89	1.22E+08	1.09E+08	89	95	
LGC023T	10.0	1.56E+10	1.37E+10	88	1.54E+08	1.36E+08	88	93	
LGC024N	9.8	1.49E+10	1.33E+10	89	1.48E+08	1.31E+08	89	94	
LGC024T	9.9	1.20E+10	1.07E+10	89	1.19E+08	1.06E+08	89	95	
LGC025T	8.7	1.37E+10	1.22E+10	89	1.36E+08	1.21E+08	89	95	
LGC026N	9.0	1.53E+10	1.31E+10	86	1.51E+08	1.30E+08	86	95	
LGC026T	8.4	1.40E+10	1.22E+10	88	1.38E+08	1.21E+08	88	95	
LGC027N	9.7	1.39E+10	1.23E+10	89	1.37E+08	1.22E+08	89	95	
LGC027T	7.3	1.17E+10	1.05E+10	90	1.15E+08	1.04E+08	90	96	
LGC028N	9.1	1.17E+10	1.05E+10	89	1.16E+08	1.04E+08	89	95	
LGC028T	7.6	1.54E+10	1.36E+10	88	1.53E+08	1.34E+08	88	95	
LGC029T	9.2	1.41E+10	1.24E+10	88	1.39E+08	1.23E+08	88	93	
LGC030N	8.1	1.21E+10	1.08E+10	89	1.20E+08	1.06E+08	89	94	
LGC030T	9.0	1.39E+10	1.24E+10	89	1.38E+08	1.23E+08	89	95	
LGC031N	8.0	1.26E+10	1.12E+10	89	1.25E+08	1.11E+08	89	94	
LGC031T	8.5	1.59E+10	1.41E+10	89	1.57E+08	1.40E+08	89	94	
LGC032T	8.7	1.45E+10	1.25E+10	86	1.44E+08	1.24E+08	86	92	
LGC033N	9.6	1.21E+10	1.08E+10	89	1.20E+08	1.07E+08	89	95	
LGC033T	9.1	1.29E+10	1.15E+10	89	1.28E+08	1.14E+08	89	95	
LGC034T	9.7	1.64E+10	1.47E+10	89	1.62E+08	1.45E+08	89	94	
LGC035T	10.0	1.54E+10	1.36E+10	88	1.52E+08	1.34E+08	88	94	
LGC036T	7.2	1.34E+10	1.18E+10	88	1.33E+08	1.17E+08	88	92	
LGC037N	8.6	9.78E+09	8.57E+09	88	9.69E+07	8.49E+07	88	95	
LGC037T	9.7	1.37E+10	1.22E+10	89	1.36E+08	1.20E+08	89	95	
LGC038T	8.6	1.14E+10	9.51E+09	83	1.13E+08	9.41E+07	83	91	
LGC039T	8.9	1.33E+10	1.19E+10	90	1.32E+08	1.18E+08	90	96	
LGC040T	9.0	1.69E+10	1.50E+10	89	1.68E+08	1.49E+08	89	94	
LGC041T	7.2	1.43E+10	1.24E+10	87	1.41E+08	1.23E+08	87	92	
LGC042T	9.5	1.02E+10	8.85E+09	87	1.01E+08	8.76E+07	87	93	
LGC043N	9.6	1.31E+10	1.17E+10	89	1.30E+08	1.15E+08	89	96	
LGC043T	9.8	1.36E+10	1.20E+10	88	1.34E+08	1.19E+08	88	95	
LGC044T	8.2	1.45E+10	1.32E+10	91	1.44E+08	1.30E+08	91	96	
LGC045T	9.6	1.38E+10	1.23E+10	89	1.37E+08	1.22E+08	89	96	
LGC046T	9.8	1.51E+10	1.33E+10	88	1.49E+08	1.32E+08	88	94	
LGC047T	7.8	1.77E+10	1.53E+10	86	1.75E+08	1.51E+08	86	92	

LGC048T	9.5	1.76E+10	1.54E+10	88	1.74E+08	1.52E+08	88	92	
LGC049N	9.2	1.11E+10	9.99E+09	90	1.10E+08	9.90E+07	90	96	
LGC049T	9.7	1.39E+10	1.22E+10	88	1.37E+08	1.21E+08	88	95	
LGC050N	9.1	1.49E+10	1.32E+10	89	1.47E+08	1.31E+08	89	96	
LGC050T	9.5	1.16E+10	1.02E+10	88	1.15E+08	1.01E+08	88	95	
LGC051N	9.1	1.49E+10	1.32E+10	89	1.48E+08	1.31E+08	89	95	
LGC051T	8.5	1.14E+10	1.02E+10	90	1.12E+08	1.01E+08	90	96	
LGC052T	10.0	1.30E+10	1.14E+10	88	1.28E+08	1.13E+08	88	93	
LGC053N	8.2	1.29E+10	1.15E+10	90	1.28E+08	1.14E+08	90	94	N[3]
LGC053T	8.2	1.39E+10	1.24E+10	89	1.37E+08	1.22E+08	89	94	N
LGC054T	9.0	1.20E+10	1.04E+10	87	1.19E+08	1.03E+08	87	91	
LGC055N	9.1	1.36E+10	1.18E+10	87	1.35E+08	1.17E+08	87	95	
LGC055T	8.2	1.55E+10	1.36E+10	88	1.53E+08	1.34E+08	88	96	
LGC056N	8.8	1.17E+10	1.03E+10	88	1.15E+08	1.02E+08	88	95	
LGC056T	7.9	1.37E+10	1.21E+10	89	1.36E+08	1.20E+08	89	96	
LGC057T	8.1	1.33E+10	1.19E+10	90	1.32E+08	1.18E+08	90	96	
LGC058N	8.9	1.18E+10	1.05E+10	89	1.17E+08	1.04E+08	89	96	
LGC058T	7.3	1.41E+10	1.26E+10	89	1.40E+08	1.25E+08	89	96	
LGC059T	10.0	1.48E+10	1.28E+10	86	1.46E+08	1.26E+08	86	93	
LGC060T	10.0	1.30E+10	1.14E+10	87	1.29E+08	1.13E+08	87	93	
LGC061T	8.8	1.40E+10	1.25E+10	89	1.38E+08	1.23E+08	89	94	
LGC062T	10.0	1.33E+10	1.17E+10	88	1.31E+08	1.16E+08	88	95	
LGP001T	10.0	1.61E+10	1.37E+10	85	1.60E+08	1.36E+08	85	92	
LGP002T	9.5	1.27E+10	1.12E+10	88	1.26E+08	1.11E+08	88	95	
LGP003T	9.1	1.32E+10	1.16E+10	88	1.31E+08	1.15E+08	88	93	
LGP004T	8.7	1.11E+10	8.50E+09	77	1.10E+08	8.41E+07	77	94	
LGP005T	10.0	1.44E+10	1.26E+10	87	1.43E+08	1.24E+08	87	94	
LGP006T	7.8	2.73E+10	2.45E+10	90	2.70E+08	2.42E+08	90	96	
LGP007T	8.6	1.53E+10	1.35E+10	88	1.52E+08	1.34E+08	88	94	
LGP008T	8.3	1.39E+10	1.24E+10	89	1.38E+08	1.23E+08	89	94	
LGP009T	10.0	1.15E+10	1.02E+10	89	1.14E+08	1.01E+08	89	93	
LGP010T	9.9	1.63E+10	1.45E+10	89	1.61E+08	1.44E+08	89	94	
LGP011T	10.0	1.39E+10	1.19E+10	86	1.38E+08	1.18E+08	86	92	
LGP012T	9.5	1.06E+10	9.12E+09	86	1.05E+08	9.03E+07	86	92	
LGP013T	9.0	1.31E+10	1.14E+10	87	1.30E+08	1.13E+08	87	93	

LGP014T	10.0	1.37E+10	1.17E+10	85	1.35E+08	1.15E+08	85	92	
LGP015T	10.0	1.06E+10	9.41E+09	89	1.05E+08	9.31E+07	89	93	
LGP016N	8.5	1.41E+10	1.26E+10	89	1.40E+08	1.25E+08	89	94	
LGP016T	9.0	1.44E+10	1.26E+10	87	1.43E+08	1.25E+08	87	94	
LGP017N	7.7	1.27E+10	1.13E+10	89	1.26E+08	1.12E+08	89	94	
LGP017T	8.7	1.36E+10	1.21E+10	89	1.35E+08	1.20E+08	89	94	
LGP018T	9.6	1.54E+10	1.37E+10	89	1.52E+08	1.36E+08	89	95	
LGP019T	8.8	1.82E+10	1.61E+10	89	1.80E+08	1.60E+08	89	94	
LGP020T	9.4	1.13E+10	9.70E+09	86	1.12E+08	9.60E+07	86	93	
LGP021T	9.9	1.62E+10	1.44E+10	89	1.60E+08	1.42E+08	89	94	
LGP022T	8.5	1.44E+10	1.27E+10	88	1.43E+08	1.26E+08	88	93	
LGP023T	9.5	1.67E+10	1.48E+10	89	1.65E+08	1.47E+08	89	95	
LGP024T	10.0	1.26E+10	1.09E+10	87	1.25E+08	1.08E+08	87	92	
LGP025T	10.0	1.12E+10	9.93E+09	89	1.10E+08	9.83E+07	89	95	
LGP026T	10.0	1.33E+10	1.17E+10	88	1.32E+08	1.16E+08	88	95	
LGP027T	8.9	1.19E+10	1.06E+10	89	1.18E+08	1.05E+08	89	95	
LGP028T	9.9	1.12E+10	9.89E+09	88	1.11E+08	9.79E+07	88	93	
LGP029N	7.7	1.25E+10	1.11E+10	89	1.23E+08	1.10E+08	89	95	
LGP029T	8.3	1.26E+10	1.12E+10	89	1.25E+08	1.11E+08	89	95	
LGP030T	8.7	1.78E+10	1.60E+10	89	1.77E+08	1.58E+08	89	94	
LGP031T	9.4	1.13E+10	9.80E+09	86	1.12E+08	9.70E+07	86	92	
LGP032N	9.5	1.05E+10	9.39E+09	89	1.04E+08	9.30E+07	89	94	N
LGP032T	10.0	1.31E+10	1.15E+10	88	1.30E+08	1.14E+08	88	94	N
LGP033N	9.1	9.36E+09	8.28E+09	88	9.27E+07	8.20E+07	88	94	
LGP033T	9.2	1.60E+10	1.43E+10	89	1.59E+08	1.41E+08	89	95	
LGP034N	9.0	1.28E+10	1.13E+10	88	1.26E+08	1.11E+08	88	94	
LGP034T	9.4	1.25E+10	1.10E+10	88	1.24E+08	1.09E+08	88	94	
LGP035T	8.3	1.40E+10	1.26E+10	89	1.39E+08	1.24E+08	89	94	
LGP036T	8.5	1.28E+10	1.15E+10	89	1.27E+08	1.13E+08	89	95	
LGP037T	9.2	1.36E+10	1.18E+10	87	1.35E+08	1.17E+08	87	92	
LGP038T	8.9	1.19E+10	1.05E+10	88	1.18E+08	1.04E+08	88	95	
LGP039T	8.1	1.49E+10	1.32E+10	88	1.48E+08	1.31E+08	88	95	
LGP040T	10.0	1.30E+10	1.14E+10	88	1.29E+08	1.13E+08	88	95	
LGP041T	9.1	1.44E+10	1.28E+10	89	1.43E+08	1.27E+08	89	94	
LGP042T	10.0	1.69E+10	1.50E+10	88	1.68E+08	1.48E+08	88	95	
LGP043T	8.8	1.44E+10	1.24E+10	86	1.43E+08	1.23E+08	86	94	

LGP044T	9.8	1.32E+10	1.14E+10	87	1.31E+08	1.13E+08	87	92	
LGP045T	8.1	1.52E+10	1.36E+10	89	1.51E+08	1.34E+08	89	95	
LGP046T	9.5	1.56E+10	1.35E+10	86	1.55E+08	1.33E+08	86	92	
LGP047T	8.9	1.63E+10	1.41E+10	86	1.62E+08	1.39E+08	86	92	
LGP048T	9.8	1.36E+10	1.18E+10	86	1.35E+08	1.16E+08	86	92	
LGP049T	6.1	8.78E+09	7.58E+09	86	8.70E+07	7.51E+07	86	92	
LGP050T	7.4	1.18E+10	1.04E+10	88	1.17E+08	1.03E+08	88	94	
LGP051T	7.1	1.04E+10	9.29E+09	89	1.03E+08	9.19E+07	89	94	
LGP052N	8.7	1.26E+10	1.11E+10	88	1.25E+08	1.10E+08	88	95	
LGP052T	9.6	1.29E+10	1.14E+10	88	1.27E+08	1.12E+08	88	95	
LGP053T	8.1	1.59E+10	1.41E+10	89	1.57E+08	1.40E+08	89	94	
LGP054N	9.6	1.24E+10	1.11E+10	90	1.22E+08	1.10E+08	90	95	N
LGP054T	8.8	1.50E+10	1.33E+10	89	1.48E+08	1.31E+08	89	95	N
LGP055T	7.6	1.43E+10	1.28E+10	89	1.42E+08	1.26E+08	89	95	
LGP056N	7.9	1.37E+10	1.22E+10	89	1.35E+08	1.20E+08	89	95	
LGP056T	10.0	1.41E+10	1.25E+10	88	1.40E+08	1.24E+08	88	95	
LGP057T	8.2	1.37E+10	1.22E+10	89	1.36E+08	1.21E+08	89	94	
LGP058N	9.6	1.44E+10	1.28E+10	89	1.43E+08	1.27E+08	89	95	
LGP058T	10.0	1.21E+10	1.08E+10	89	1.20E+08	1.07E+08	89	95	
LGP059N	8.3	1.41E+10	1.26E+10	89	1.39E+08	1.24E+08	89	95	
LGP059T	10.0	1.72E+10	1.53E+10	89	1.70E+08	1.51E+08	89	95	
LGP060N	5.7	1.51E+10	1.36E+10	90	1.50E+08	1.35E+08	90	95	
LGP060T	9.0	1.23E+10	1.09E+10	89	1.22E+08	1.08E+08	89	95	
LGP061T	7.5	1.32E+10	1.18E+10	89	1.31E+08	1.17E+08	89	94	
LGP062N	9.1	1.52E+10	1.35E+10	89	1.50E+08	1.34E+08	89	95	
LGP062T	10.0	1.50E+10	1.33E+10	89	1.48E+08	1.32E+08	89	95	
LGP063N	5.8	1.15E+10	1.02E+10	89	1.14E+08	1.01E+08	89	95	
LGP063T	9.3	1.35E+10	1.17E+10	87	1.33E+08	1.16E+08	87	95	
LGP064T	8.1	1.48E+10	1.32E+10	89	1.47E+08	1.30E+08	89	94	
LGP065T	9.8	1.43E+10	1.26E+10	88	1.41E+08	1.25E+08	88	94	
LGP066N	8.9	1.18E+10	1.05E+10	89	1.17E+08	1.04E+08	89	95	
LGP066T	7.5	1.25E+10	1.11E+10	89	1.24E+08	1.10E+08	89	95	
LGP067N	9.4	1.57E+10	1.40E+10	89	1.55E+08	1.39E+08	89	95	
LGP067T	8.9	1.59E+10	1.42E+10	89	1.57E+08	1.40E+08	89	95	
LGP068T	8.8	1.26E+10	1.13E+10	89	1.25E+08	1.12E+08	89	95	
LGP069N	9.9	1.25E+10	1.11E+10	89	1.23E+08	1.10E+08	89	95	

LGP069T	10.0	1.31E+10	1.17E+10	89	1.30E+08	1.16E+08	89	95	
LGP070N	8.0	1.35E+10	1.20E+10	89	1.34E+08	1.19E+08	89	95	
LGP070T	10.0	1.37E+10	1.20E+10	88	1.35E+08	1.19E+08	88	95	
LGP071N	5.6	1.19E+10	1.06E+10	89	1.18E+08	1.05E+08	89	95	
LGP071T	9.8	1.16E+10	1.04E+10	90	1.15E+08	1.03E+08	90	95	
LGP072T	9.4	9.75E+09	8.65E+09	89	9.65E+07	8.57E+07	89	95	
LGP073T	9.2	1.40E+10	1.24E+10	88	1.39E+08	1.22E+08	88	95	
LGP074T	10.0	1.65E+10	1.46E+10	88	1.64E+08	1.45E+08	88	94	
LGP075N	7.9	1.55E+10	1.40E+10	90	1.54E+08	1.39E+08	90	95	
LGP075T	9.9	1.41E+10	1.25E+10	89	1.39E+08	1.24E+08	89	95	
LGP076T	9.8	1.55E+10	1.38E+10	89	1.54E+08	1.37E+08	89	95	
LGP077T	8.9	1.19E+10	1.06E+10	89	1.18E+08	1.05E+08	89	94	
LGP078T	9.9	1.30E+10	1.12E+10	86	1.29E+08	1.10E+08	86	92	
LGP079N	8.0	1.37E+10	1.21E+10	89	1.35E+08	1.20E+08	89	94	
LGP079T	9.1	2.01E+10	1.76E+10	88	1.99E+08	1.74E+08	88	95	
LGP080T	9.1	1.38E+10	1.22E+10	89	1.37E+08	1.21E+08	89	95	
LGP081N	9.4	1.55E+10	1.37E+10	88	1.54E+08	1.36E+08	88	94	
LGP081T	9.5	1.46E+10	1.28E+10	87	1.45E+08	1.26E+08	87	95	
LGP082N	8.9	1.52E+10	1.33E+10	88	1.50E+08	1.32E+08	88	95	
LGP082T	9.7	1.50E+10	1.33E+10	89	1.49E+08	1.32E+08	89	95	
LGP083N	6.8	1.03E+10	9.14E+09	89	1.02E+08	9.05E+07	89	95	
LGP083T	9.5	1.23E+10	1.11E+10	90	1.22E+08	1.09E+08	90	95	
LGP084T	6.3	1.34E+10	1.20E+10	90	1.33E+08	1.19E+08	90	95	
LGP085T	10.0	1.33E+10	1.14E+10	86	1.32E+08	1.13E+08	86	92	
LGP086T	8.9	1.49E+10	1.33E+10	89	1.47E+08	1.32E+08	89	95	
LGP087T	10.0	1.80E+10	1.59E+10	89	1.78E+08	1.58E+08	89	94	
LGP088T	9.9	1.53E+10	1.36E+10	89	1.51E+08	1.34E+08	89	93	

[1]RIN: RNA integrity number

[2]Q20: Lower base call accuracy of 99%

[3]N: Not included in further analysis

Table S3. Fusion genes identified in Korean patients with colon cancer

Algorithm	Patient ID	Stage	Type	Frame	Discordant paired-end reads	Spanning reads	Distance	Donor gene	Full name	FPKM	Band	Location	Strand	Exon break	NT Seq before break	Domain	AA seq before break
GFP,FusionMap	LGP088T	III	Intra	In	27	153	2620933	TPM3	Tropomyosin 3	59	1q21.3	chr1:154142876	-	8	G--	Coiled coil	D
GFP,FusionMap, deFuse	LGC012T	III	Intra	In	25	132	2620933	TPM3	Tropomyosin 3	94	1q21.3	chr1:154142876	-	8	G--	Coiled coil	D
								TPM3	Tropomyosin 3	94	1q21.3	chr1:154142876	-	8	G--	Coiled coil	D
GFP,FusionMap, deFuse	LGC026T	II	Intra	In	11	15	675664	LMNA	Lamin A/C	145	1q22	chr1:156105740	+	6	C--	Coil2	R
GFP,FusionMap	LGC007T	III	Intra	In	21	197	771740	PTPRK	Protein tyrosine phosphatase, receptor type, K	17	6q22.33	chr6:128841404	-	1	G--	-	G
GFP,FusionMap	LGC054T	III	Intra	In	36	140	771740	PTPRK	Protein tyrosine phosphatase, receptor type, K	15	6q22.34	chr6:128505577	-	7	G--	-	G
GFP,FusionMap, deFuse	LGC030T	III	Intra	In	56	374	2667510	NAGLU	N-acetylglucosaminidase, alpha	255	17q21.2	chr17:40693224	+	4,5	G--	-	A
GFP,FusionMap, deFuse	LGP047T	II	Intra	In	45	206	1020112	GTF3A	general transcription factor IIIA	641	13q12.2	chr13:27999075	+	1	GAG	Zinc finger	E
GFP,FusionMap	LGP024T	II	Intra	In	24	98	55463	RAD51AP1	RAD51 associated protein 1	7	12p13.32	chr12:4662255	+	8	AAG	-	K
GFP,FusionMap	LGP031T	II	Intra	In	17	53	28829615	RASA1	RAS p21 protein activator	11	5q14.3	chr5:86564807	+	1	CA-	-	Q

Algorithm	Patient ID	Stage	Type	Frame	Discordant paired-end reads	Spanning reads	Distance	Acceptor	Full name	FPKM	Band	Location	Strand	Exon break	NT seq after break	Domain	AA after break
GFP,FusionMap	LGP088T	III	Intra	In	27	153	2620933	NTRK1	Neurotrophic tyrosine kinase, receptor, type 1	31	1q23.1	chr1:156844363	+	9	-AC	Extracellular	D
GFP,FusionMap ,deFuse	LGC012T	III	Intra	In	25	132	2620933	NTRK1	Neurotrophic tyrosine kinase, receptor, type 1	20	1q23.1	chr1:156845312	+	11	-GC	Extracellular	G
								NTRK1	Neurotrophic tyrosine kinase, receptor, type 1	20	1q23.1	chr1:156845872	+	12	-GT	Extracellular	G

GFP,FusionMap ,deFuse	LGC0 26T	II	Int ra	In	11	15	6756 64	NTRK 1	Neurotrophic tyrosine kinase, receptor, type 1	32	1q23. 1	chr1:1568 45312	+	11	- G C	Extracellular	R
GFP,FusionMap	LGC0 07T	II I	Int ra	In	21	197	7717 40	RSPO3	R-spondin 3	31	6q22. 33	chr6:1274 69793	+	2	- T G	Thrombospondin type-1 (TSP1)	M
GFP,FusionMap	LGC0 54T	II I	Int ra	In	36	140	7717 40	RSPO3	R-spondin 3	37	6q22. 34	chr6:1274 69793	+	2	- T G	Thrombospondin type-1 (TSP2)	M
GFP,FusionMap ,deFuse	LGC0 30T	II I	Int ra	In	56	374	2667 510	IKZF3	IKAROS family zinc finger 3 (Aiolos)	55	17q1 2	chr17:379 22746	-	6, 7, 8	- G T	Zinc finger	V
GFP,FusionMap ,deFuse	LGP0 47T	II	Int ra	In	45	206	10201 12	CDK8	Cyclin-dependent kinase 8	93	13q1 2.13	chr13:269 23209	+	3	TT A	Protein kinase domain	L
GFP,FusionMap	LGP0 24T	II	Int ra	In	24	98	5546 3	AKAP 3	A kinase (PRKA) anchor protein 3	18	12p1 3.32	chr12:473 7971	-	4	G T C	-	D
GFP,FusionMap	LGP0 31T	II	Int ra	In	17	53	2882 9615	LOC64 4100	ADP-ribosylation factor-like 14 effector protein-like (ARL14EPL)	19	5q23. 1	chr5:1153 94422	+	3	-- A	-	K

Table S4. Correlation between TrkA protein expression and NTRK1 fusion

Sample ID	Cytoplasmic TrkA expression	NTRK1 fusion	*p*-value[1]
	IHC	FISH	
Colon_50_FISH01	Strong	Positive	0.0192
Colon_50_FISH02	Strong	Negative	
Colon_50_FISH03	Strong	Not determined	
Colon_50_FISH04	Strong	Positive	
Colon_50_FISH05	Strong	Negative	
Colon_50_FISH06	Moderate	Positive	
Colon_50_FISH07	Moderate	Negative	
Colon_50_FISH08	Weak	Negative	
Colon_50_FISH09	Weak	Negative	
Colon_50_FISH10	Weak	Negative	
Colon_50_FISH11	Negative	Negative	
Colon_50_FISH12	Negative	Negative	
Colon_50_FISH13	Negative	Negative	
Colon_50_FISH14	Negative	Negative	
Colon_50_FISH15	Negative	Negative	

[1]p-value: One-sided chi-square test between TrkA strong/moderate to weak/negative tumors with split NTRK1 FISH signals
